# *Platycladus orientalis* Leaf-Derived Exosome-like Nanovesicles Delivered by Hydrogel Microneedles Restore Follicular Immune Homeostasis and Promote Hair Regeneration in Androgenetic Alopecia

**DOI:** 10.3390/biom16101505

**Published:** 2026-10-15

**Authors:** Xueying Shi, Yueming Wang, Lingai Meng, Yuxia Wei, Guanglu Yin, Qian Xue, Qingxin Shang, Fanyue Zhou, Qianqian Luo, Yanlian Xiong

**Affiliations:** 1 Xu Rongxiang Regenerative Medicine Research Center, Shandong Medical and Pharmaceutical University, Yantai 264003, China; 2 Laboratory of Pathogenic Biology and Immunology, Shandong Medical and Pharmaceutical University, Yantai 264003, China; 3 Shanghai MEBO Life Science & Technology Co., Ltd., Shanghai 201321, China; 4 The Second Clinical Medical College, Shandong Medical and Pharmaceutical University, Yantai 264003, China

**Keywords:** androgenetic alopecia, *Platycladus orientalis*, plant-derived nanovesicles, hair follicle regeneration, Wnt/β-catenin signaling pathway, transdermal drug delivery

## Abstract

Androgenetic alopecia (AGA) is the most prevalent form of hair loss, yet current therapies offer limited efficacy and fail to restore the disrupted follicular immune microenvironment. Here, we developed a bioactive hydrogel microneedle system (P-MN) in which *Platycladus orientalis leaf*-derived exosome-like nanovesicles (Plao-ELNs) were encapsulated within a methacrylated hyaluronic acid/cactus polysaccharide (HAMA/CP) matrix for transdermal AGA therapy. The HAMA/CP hydrogel was evaluated for biocompatibility, antibacterial activity, and effects on the migration of dermal papilla cells (DPCs) in vitro; P-MN was assessed for hair regrowth, follicular architecture, and anagen induction in a murine AGA model; and the underlying mechanisms were examined using transcriptomics, flow cytometry, and molecular assays. The HAMA/CP hydrogel showed good biocompatibility and antibacterial activity, and enhanced the migration of DPCs, while enabling sustained intradermal retention of Plao-ELNs. In vivo, P-MN accelerated hair regrowth, restored follicular architecture, and increased the proportion of hair follicles in the anagen phase. Mechanistically, Plao-ELNs suppressed aberrant T-cell activation and reduced IL-17A secretion, thereby inhibiting NF-κB-mediated DKK1 expression and reactivating Wnt/β-catenin signaling in DPCs to promote hair follicle regeneration. The HAMA/CP matrix potentiated efficacy through antibacterial protection and prolonged local delivery, supporting P-MN as a promising strategy for AGA treatment.

## 1. Introduction

Androgenetic alopecia (AGA) is the most prevalent form of hair loss worldwide and is characterized by progressive miniaturization of hair follicles and shortening of the anagen phase, both driven primarily by androgen-dependent signaling [1]. Epidemiological data indicate that AGA affects approximately 50% of men by 50 years of age, imposing a substantial psychosocial burden [2]. Current first-line pharmacotherapies remain limited to topical minoxidil and oral finasteride. However, because AGA is a multifactorial disorder involving microinflammation and oxidative stress, these monotherapies rarely achieve satisfactory hair regrowth [3]. Their clinical utility is further compromised by adverse effects, such as scalp irritation and systemic hormonal imbalance, and by the poor compliance associated with long-term daily application [4]. There is therefore an urgent need for next-generation therapeutic systems that combine high target specificity, minimal toxicity, and sustained efficacy.

Recent evidence has reframed AGA pathogenesis as an immune-mediated inflammatory process in which disruption of the Th17/Treg balance plays a pivotal role [5]. The hair follicle is an immune-privileged organ; in the AGA microenvironment, however, this privilege is compromised, leading to an aberrant shift toward Th17-mediated responses and a concomitant reduction in regulatory T cells (Tregs) [6]. Clinical investigations have documented substantial infiltration of CD4^+^IL-17A^+^ Th17 cells into the dermis of the scalp in patients with AGA, with the infiltrate localized predominantly to perifollicular regions [7], together with a marked reduction in Tregs that skews the Th17/Treg equilibrium [8]. Although this imbalance was first characterized in alopecia areata, it has since been robustly confirmed in AGA [9]. Restoration of the Th17/Treg equilibrium has therefore emerged as a critical therapeutic strategy for reversing follicular miniaturization.

To address these challenges, plant-derived exosome-like nanovesicles (PELNs) have emerged as biogenic platforms for regenerative medicine [10,11]. Among botanical candidates, *Platycladus orientalis* has a long history of use in traditional medicine for promoting hair growth. Modern pharmacological studies have begun to rationalize this traditional use, attributing the hair-restorative effects of *P. orientalis* extracts to activation of the Wnt/β-catenin pathway and to alleviation of oxidative stress [12]. Beyond the ethnopharmacological profile of their source plants, nanovesicles derived from medicinal plants exhibit intrinsic immunomodulatory activity and can deliver endogenous flavonoids and functional small RNAs to recipient cells [13,14,15]. A recent study demonstrated that *P. orientalis*-derived extracellular vesicles (PO-EVs), co-delivered with minoxidil-loaded nanoparticles via dissolving microneedles, exert antioxidant, anti-inflammatory, and pro-angiogenic effects that facilitate the telogen-to-anagen transition [16]. Nevertheless, such synergistic strategies have been evaluated mainly in the context of innate inflammatory and angiogenic mechanisms; whether *P. orientalis leaf*-derived exosome-like nanovesicles (Plao-ELNs) can modulate adaptive immunity remains unexplored and, in particular, whether they can reverse androgen-driven follicular miniaturization by rebalancing the Th17/Treg axis remains to be determined.

The therapeutic efficacy of topically applied PELNs is further constrained by the stratum corneum, which restricts penetration into the deep follicular niche. Hydrogel microneedles (MNs) offer a minimally invasive means of circumventing this barrier [17,18]. Recent pilot studies have integrated PELNs into microneedle systems and have demonstrated improved stability and transdermal permeation of botanical bioactives [19]. By creating transient microchannels, these MNs enable the sustained, localized release of encapsulated cargo directly into the perifollicular environment, thereby maximizing the biological potency of the delivered agents [20].

In this study, we developed a hydrogel microneedle system (P-MN) in which Plao-ELNs serve as the therapeutic payload encapsulated within a methacrylated hyaluronic acid/cactus polysaccharide (HAMA/CP) matrix. We systematically evaluated the efficacy of P-MN in reversing hair loss using a murine AGA model and in vitro cellular assays. By integrating transcriptomic profiling with flow cytometry and molecular validation, we investigated the underlying mechanisms, focusing specifically on how P-MN modulates T-cell differentiation and restores the Th17/Treg equilibrium within the follicular microenvironment. Furthermore, we examined the downstream signaling crosstalk that drives the reactivation of dermal papilla cells (DPCs). Our findings identify P-MN as a promising immunomodulatory strategy for AGA and provide mechanistic insight into how plant-derived exosome-like nanovesicles restore follicular immune homeostasis to promote hair regrowth.

## 2. Materials and Methods

### 2.1. Extraction and Characterization of Plao-ELNs

Fresh leaves of *P. orientalis* were collected from Zaozhuang (Shandong, China). Healthy mature leaves of *P. orientalis* were harvested at the full-grown vegetative stage, and leaves with consistent developmental status were selected for extraction. To isolate and purify Plao-ELNs, a differential ultracentrifugation protocol was employed. Briefly, fresh *P. orientalis* leaves were rinsed twice with deionized water and mixed with phosphate-buffered saline (PBS) at 4 °C. The mixture was placed in a homogenizer to grind the leaves and extract the juice, followed by sequential centrifugation at 4 °C: First, it was centrifuged at 5000× *g* for 30 min, then at 8000× *g* for 30 min, followed by centrifugation at 10,000× *g* for 30 min, until no visible sediment remained. Subsequently, the resulting supernatant was centrifuged at 100,000× *g* for 90 min at 4 °C using an ultracentrifuge (Optima XE-100, Beckman Coulter, Brea, CA, USA). The pellet was collected, resuspended in PBS, and filtered through a 0.22 μm membrane filter. The resulting Plao-ELNs were aliquoted and stored at −80 °C until use.

For morphological evaluation, Plao-ELNs were negatively stained and observed via transmission electron microscopy (TEM) (Hitachi JEM-1400, Tokyo, Japan). The particle size distribution and concentration were further determined by nanoparticle tracking analysis (NTA) using a Particle Metrix system (Particle Metrix GmbH, Inning am Ammersee, Germany). The zeta potential of Plao-ELNs was characterized using a zeta potential analyzer (NanO ZS, Worcestershire, UK). Briefly, Plao-ELNs samples were diluted to a suitable concentration with PBS and carefully loaded into the capillary measurement cell to avoid air bubbles. The measurement temperature was maintained at 25 °C. All samples were detected in triplicate. The total protein concentration of Plao-ELNs was quantified using a BCA protein assay kit (PC0020, Solarbio, Beijing, China) according to the manufacturer’s instructions.

### 2.2. SDS-PAGE Analysis of Plao-ELN Proteins

To analyze the protein composition, Plao-ELN samples were lysed using SDT buffer (4% SDS, 100 mM Tris-HCl, pH 7.6) for total protein extraction. The protein concentration was standardized, and 20 μg of protein was mixed with loading buffer and boiled at 95 °C for 5 min. The protein lysates were then separated via SDS-PAGE using 4–20% precast gradient gels at a constant voltage of 120 V for 60 min. After electrophoresis, the protein bands were visualized through staining with Coomassie Brilliant Blue R-250 (C8430, Solarbio, China) and imaged using a gel documentation system (5200, Tanon, Shanghai, China).

### 2.3. Metabolomic Composition Analysis of Plao-ELNs

The metabolite profile of the Plao-ELNs was characterized using liquid chromatography–tandem mass spectrometry (LC-MS/MS), which was conducted by Shanghai Luming Biotechnology Co., Ltd. (Shanghai, China) Chromatographic separation was performed using an ACQUITY UPLC HSS T3 system. The mobile phase comprised solvent A (0.1% *v*/*v* formic acid in water,) and solvent B (acetonitrile). A programmed gradient elution was performed at a flow rate of 0.35 mL/min, with the column temperature maintained at 45 °C.

Mass spectrometry was performed using a Thermo-Orbitrap-QE HF instrument (Thermo Fisher Scientific, Waltham, MA, USA) operating in both positive and negative electrospray ionization (ESI) modes. Data acquisition was carried out in data-dependent acquisition (DDA) mode, employing a Full MS/dd-MS2 (Top 10) scanning strategy. The resolution was set at 60,000 for full MS scans, and 15,000 for MS/MS scans to ensure high-mass accuracy and sensitivity.

### 2.4. Synthesis and Characterization of Methacrylated Hyaluronic Acid (HAMA)

Methacrylated hyaluronic acid (HAMA) was synthesized via the chemical modification of hyaluronic acid (HA, H823435, Macklin, North Vancouver, BC, USA) with methacrylic anhydride (MA, Shanghai Yuanye Bio-Technology, Shanghai, China) according to previously established protocols [21]. Briefly, 1.0 g of HA was dissolved in 100 mL of deionized water and stirred for 6 h to ensure complete dissolution. Subsequently, 5 mL of MA was added dropwise to the HA solution. Throughout the reaction, the pH of the mixture was maintained between 8.5 and 9.0 by the periodic addition of a sodium hydroxide (NaOH) solution. The reaction was allowed to proceed for 24 h at 4 °C under continuous stirring. The resulting mixture was then transferred to a dialysis membrane and dialyzed against deionized water for 3 days (with the dialysis medium replaced every 4 h) to remove unreacted MA and other impurities. The purified HAMA was obtained as a white foam after lyophilization.

### 2.5. Extraction and Purification of Cactus Polysaccharides (CP)

Fresh cactus stems were sliced and rapidly frozen at −80 °C until completely solidified. The frozen samples were thoroughly dried in a freeze dryer (Alphal-2LDplus, Osterode am Harz, Germany), ground with a high-speed grinder, and sieved to obtain homogeneous particles. Cactus powder was mixed with ultrapure water at a solid-to-liquid ratio of 1:30 (*w*/*v*) and soaked for 30 min. The mixture was subjected to ultrasonic treatment for 2 h in a 42 °C constant-temperature water bath using an ultrasonic cleaner with intermittent stirring. Subsequently, the supernatant was collected by centrifugation, and anhydrous ethanol was added to fully precipitate polysaccharides. The ethanol-precipitated polysaccharides were redissolved in an appropriate volume of ultrapure water, followed by dialysis and freeze-drying to yield CP.

### 2.6. Preparation and Characterization of HAMA/CP Composite Hydrogels

To fabricate the composite hydrogel, HAMA and extracted CP were dissolved in PBS to form a precursor solution. The photoinitiator I2959 (2-hydroxy-1-[4-(2-hydroxyethoxy)phenyl]-2-methylpropan-1-one; Irgacure 2959) was added. The mixture was then exposed to 365 nm UV light for 30 s to induce photo-crosslinking, resulting in the formation of the HAMA/CP hydrogel. The cross-sectional morphology and internal porous microarchitecture of the lyophilized HAMA/CP hydrogel were characterized via scanning electron microscopy (SEM) (EVO LS15, Zeiss, Jena, Germany).

To confirm successful chemical modification, Fourier transform infrared (FTIR) spectroscopy was performed using a Frontier Mid-IR FTIR spectrometer (Perkin-Elmer, Waltham, MA, USA). The spectra of native HA, MA, CP, and the synthesized HAMA/CP hydrogel were recorded across a wavenumber range of 4000–500 cm^−1^.

### 2.7. Hemolysis Assay

Red blood cells (RBCs) were collected from fresh mouse blood (5 mL) by centrifugation. The RBCs were washed with PBS and finally resuspended in PBS to prepare a 2% suspension. The freshly prepared RBCs suspension (1 mL) was added into the HAMA/CP hydrogels (6 mg) in 1.5 mL EP tubes, and incubated at 37 °C for 4 h. HAMA/CP hydrogels were removed and the treated RBCs suspension was centrifuged at 2000 rpm for 5 min, and the supernatants were subjected to absorbance detection at 540 nm. PBS and 1% Triton X-100 were used as the negative and positive controls, respectively. The hemolysis ratio was calculated as follows:

$$\mathrm{Hemolysis}\left(\%\right)=\frac{A_{\mathrm{sample}}-A_{\mathrm{negative}}}{A_{\mathrm{positive}}-A_{\mathrm{negative}}}\times 100\%$$

### 2.8. Antibacterial Activity Assay

The antibacterial properties of the hydrogels were evaluated against *Escherichia coli* (ATCC 8739) and *Staphylococcus aureus* (ATCC 6538). Briefly, hydrogel extracts were prepared by incubating the HAMA and HAMA/CP hydrogels in Luria–Bertani (LB) medium. Bacterial suspensions (10^6^ CFU/mL) were inoculated with the respective hydrogel extracts or pure LB medium (serving as the control group) and incubated at 37 °C for 8 h. Subsequently, the bacterial suspensions were serially diluted and spread on LB agar plates. After overnight incubation at 37 °C, the antibacterial effects were recorded.

### 2.9. In Vitro Cell Scratch Assay

The effect of the hydrogels on the migratory capacity of DPCs was assessed using an in vitro scratch assay. DPCs were seeded into 6-well plates at a density of 1 × 10^6^ cells per well and cultured until they reached a confluent monolayer. A sterile 200 μL pipette tip was used to create a uniform linear scratch across the cell surface. After washing twice with PBS to remove cellular debris, the cells were incubated with 2% FBS DMEM supplemented with extracts from either HAMA or HAMA/CP hydrogels, whereas the control group received serum-free DMEM only. Cell migration was monitored and photographed at 0 and 24 h post-scratching using an inverted fluorescence microscope (Revolve FL, Echo Laboratories, Flagstaff, AZ, USA). The area of the denuded zone was quantified using ImageJ software (ImageJ2), and the wound closure percentage was calculated using the following formula:

$$\mathrm{Migration}\mathrm{Area}\left(\%\right)=\frac{\mathrm{Area}\left(0\;h\right)-\mathrm{Area}\left(24\;h\right)}{\mathrm{Area}(0\;h)}\times 100\%$$

### 2.10. Fabrication and Property Evaluation of P-MN

The P-MN was fabricated using a micromolding method [22]. Briefly, the Plao-ELNs were added to the HAMA/CP (10% *w*/*v* HAMA concentration) matrix and gently mixed. 0.1 mL of Plao-ELNs stock solution was added into each PDMS micromold during microneedle fabrication. Under light-shielded conditions, the respective precursors were cast into polydimethylsiloxane (PDMS) micromolds (1 cm in diameter) and degassed to ensure complete filling of the microneedle cavities. Subsequently, the MNs were subjected to 365 nm UV irradiation for 30 s for photo-crosslinking. After drying, the MNs were carefully demolded. The morphology of the MNs was characterized via SEM (EVO LS15, Zeiss). To evaluate the spatial distribution of the Plao-ELNs, DiI-labeled Plao-ELNs were encapsulated into the MNs following the same protocol, and fluorescent images were acquired using a fluorescence microscope (Echo Laboratories, Revolve FL).

### 2.11. Degradation Measurement of P-MN

The prepared P-MN was placed in PBS buffer (pH 7.2) and incubated at 37 °C. The P-MN was retrieved at preset time intervals. After wiping off excess surface liquid, photographs were acquired to visually characterize the in vitro degradation process of P-MN.

### 2.12. Skin Puncture Test of P-MN

Ex vivo full-thickness mouse skin was used to evaluate the skin puncture performance of the P-MN. Full-thickness dorsal mouse skin was excised, subcutaneous adipose tissue was removed, and the skin was washed with PBS. The P-MN was vertically placed on the skin surface and subjected to constant pressure for 1 min before removal. To determine the insertion depth, the punctured skin tissue was fixed, embedded, sectioned and stained with H&E.

### 2.13. In Vivo Fluorescence Imaging of P-MN

In vivo fluorescence imaging was performed to investigate the intradermal retention and sustained release of P-MN. Plao-ELNs were fluorescently labeled with DiI dye, and free dye was removed by purification. DiI-labeled Plao-ELNs were loaded into the MN. After removing the dorsal hair of healthy mice, the P-MN was vertically applied to the dorsal skin under constant pressure for 1 min and retained on the skin. As a control, B-MN containing PBS was administered identically on the dorsal skin of mice in the control group. The fluorescence signal intensity at the skin site was detected at predetermined time points using an in vivo animal imaging system (IVIS Spectrum, PerkinElmer, Shelton, CT, USA). The local skin retention and sustained release process of Plao-ELNs were observed to characterize the transdermal delivery performance of the MN.

### 2.14. Establishment of AGA Model

Healthy male C57BL/6 mice (6 weeks old) were purchased from Jinan Pengyue Laboratory Animal Breeding Co., Ltd. (Jinan, Shandong, China). All mice were housed in a temperature-controlled environment with a 12 h light/dark cycle and provided with ad libitum access to food and water. After one week of acclimatization, the dorsal hair of the mice was shaved with an electric clipper and further removed using a depilatory cream. Mice exhibiting no visible skin irritation or wounds were selected for the study and randomly assigned into five groups (n = 10 per group): (1) Control group, (2) AGA model group, (3) Blank microneedle group (B-MN), (4) Plao-ELN-loaded microneedle group (P-MN), and (5) Minoxidil group (MXD).

To establish the AGA model, a testosterone propionate solution was administered via daily subcutaneous injections at multiple sites within the shaved dorsal area, following a previously validated protocol [23]. For in vivo administration, one MN patch was applied to the dorsal skin of each mouse per treatment. The MN patch was gently pressed onto the shaved dorsal skin for 1 min, and the backing layer was removed after 5 min. The treatment was administered once every 3 days, on days 0, 3, 6, 9, 12, 15, and 18 after shaving, resulting in a total of seven MN applications per animal during the treatment period. In contrast, topical MXD (5%, *w*/*v*, in an ethanol/propylene glycol vehicle, 0.075 mL per mouse) was administered once daily, consistent with the conventional topical administration regimen. The treatment regimen was maintained for 28 consecutive days. Throughout the experimental period, the hair regrowth status was monitored and recorded. At the end of the treatment, the mice were euthanized via CO_2_ inhalation, followed by cervical dislocation. Skin tissues and major organs were harvested; a portion of the samples was immediately processed for transcriptomic sequencing, while the remainder was fixed in 4% paraformaldehyde or stored at −80 °C for subsequent validation. All animal experiments complied with ARRIVE 2.0 guidelines. All animal procedures were approved by the Institutional Animal Care and Use Committee of Shandong Medical and Pharmaceutical University (Approval No. 2026-010, approved on 20 January 2026).

### 2.15. Transcriptome Sequencing and Bioinformatics Analysis

Samples were sequenced by Beijing Novogene Technology Co., Ltd. (Novogene, Beijing, China). RNA was extracted from the tissues or cells using standard extraction methods, followed by rigorous quality control of the RNA samples, primarily through the Agilent 2100 Bioanalyzer to accurately assess RNA integrity. cDNA libraries were constructed and sequenced using the Illumina platform.

Raw reads were filtered and checked for sequencing error rates and GC content distribution to obtain clean reads for subsequent analysis. The clean reads were then aligned to the reference genome. HISAT2 software (version 2.2.1) was used to perform rapid and accurate alignment of clean reads to the reference genome, obtaining read mapping information for use. Gene expression quantification was performed using the featureCounts tool within the subread software.

### 2.16. Differential Expression and Functional Enrichment Analysis

Three independent biological replicates were sequenced for each experimental group (n = 3 per group). Differential expression analysis was performed between the AGA and P-MN groups using DESeq2 version 1.42.0. Genes meeting the thresholds of |log2FC| ≥ 1 and padj ≤ 0.05 were defined as differentially expressed genes (DEGs). The sequencing reads were mapped against the *Mus musculus* C57BL/6NJ reference genome (C57BL_6NJ_v1), Ensembl release 110. To further elucidate the biological functions and signaling pathways associated with these DEGs, Gene Ontology (GO) functional enrichment and Kyoto Encyclopedia of Genes and Genomes (KEGG) pathway analyses were conducted using the clusterProfiler software (version 4.8.1). A significance threshold of *p* < 0.05 was applied to identify the significantly enriched biological processes and metabolic pathways.

### 2.17. Hematoxylin and Eosin (H&E) Staining

Dorsal skin tissues harvested from each group were fixed in 4% paraformaldehyde (P1110, Solarbio) for 24 h. Following fixation, the samples were dehydrated through a graded series of ethanol, cleared in xylene, and embedded in paraffin wax. The tissues were then sectioned into 5-μm-thick sections using a microtome. After deparaffinization and rehydration, the sections were stained with hematoxylin and eosin (H&E) according to standard histological protocols. Finally, the slices were dehydrated, cleared, and mounted with a permanent medium. To assess the treatment efficacy, the histological slides were observed under an optical microscope. Two independent pathologists, blinded to the experimental groupings, evaluated the hair follicle density, morphological development, and specific hair cycle stages (anagen, catagen, or telogen).

According to the established mouse hair follicle classification system described by Müller-Röver et al. (2001) [24]. Briefly, individual hair follicles were classified according to their morphological characteristics, including hair bulb size, the position and morphology of the dermal papilla, and the overall architecture of the follicle. Hair follicles were categorized into the telogen phase, anagen phases (anagen I–VI), or catagen phases (catagen I–VIII) according to the Müller-Röver classification system. A numerical hair cycle score was then assigned to the corresponding stages, with lower scores indicating more active anagen development and higher scores indicating progression toward catagen/telogen stages.

### 2.18. SEM of Regenerated Hair Shafts

The microstructure of regenerated hair shafts was observed by SEM. The newly grown hair shafts were harvested from the dorsal skin of mice and cleaned to remove surface impurities. After gradient ethanol dehydration and sufficient drying, the hair samples were fixed flat on conductive adhesive and subjected to gold sputtering. The surface morphology, cuticle integrity and structural regularity of regenerated hair shafts were observed and compared among different groups.

### 2.19. Isolation, Culture, and Identification of DPCs

DPCs were isolated from the whisker follicles of 6-week-old male C57BL/6 mice using a combination of microdissection and collagenase digestion, as previously described [25]. Briefly, the upper lip containing the vibrissal pad was excised under anesthesia, and the dermal side was exposed under an Olympus stereomicroscope. All hair follicle bulbs were severed using micro-scissors and digested with 0.2% type I collagenase (1904MG100, BioFroxx, Einhausen, Germany) at 37 °C for 30 min. The isolated DP spheroids were resuspended in high-glucose DMEM (PM150210, Procell, Bethel, CT, USA) containing 20% fetal bovine serum (FBS, 164210, Procell) and 1% penicillin/streptomycin, and cultured in a 37 °C incubator with 5% CO_2_. By day 3, primary DPCs migrated out of the DP spheroids. By day 7, DPCs exhibited aggregate growth behavior around the DP spheroids. Passaged DPCs were cultured in DMEM supplemented with 10% FBS and 1% penicillin/streptomycin, and passaged every 3–4 days. Subsequent experiments used DPCs from the third to sixth passages.

Immunofluorescence staining was used to detect the expression of specific markers in DPCs. Cells were seeded onto cell culture slides and cultured until 70–80% confluence. The culture medium was then removed, and the cells were fixed with 4% paraformaldehyde for 20 min, permeabilized with 0.2% Triton X-100 for 10 min, and blocked with 5% bovine serum albumin for 30 min. Subsequently, the following primary antibodies were added: alkaline phosphatase (ALP) (A0514, ABclonal, Woburn, MA, USA, 1:200) and β-catenin (A19657, ABclonal, 1:100), and the samples were incubated overnight at 4 °C. The next day, fluorescently labeled secondary antibody (Dylight 488/594, A23420/A23210, Abbkine, Wuhan, China, 1:200) was added, and the samples were incubated at room temperature in the dark for 1 h. The cell nuclei were counterstained with DAPI (G1012, Servicebio, Wuhan, China) for 5 min. Fluorescent images were captured using a fluorescence microscope (Echo Laboratories, Revolve FL) to visualize the localization and expression levels of the markers.

### 2.20. Isolation and Sorting of CD4^+^ T Cells

Splenic CD4^+^ T cells were isolated from C57BL/6 mice following standardized protocols. Briefly, the spleens were minced and gently pressed through a 70 µm nylon cell strainer with the plunger of a disposable syringe, and the cell suspension was collected in pre-chilled PBS to obtain a single-cell suspension. The resulting cell suspension was centrifuged at 300× *g* for 5 min at 4 °C. Subsequently, CD4^+^ T cells were isolated using a Mag-Beads CD4^+^ T Cell Isolation Kit (MIM002N, Elabscience, Houston, TX, USA) according to the manufacturer’s instructions. The purity of the sorted CD4^+^ T cells was verified via flow cytometry prior to use in subsequent co-culture or activation experiments.

### 2.21. Enzyme-Linked Immunosorbent Assay (ELISA)

To quantitatively measure the secretion of cytokines and inflammatory factors, culture supernatants from in vitro-induced Th17 cells and tissue samples were collected separately. Cell culture supernatants were centrifuged at 1000× *g* for 10 min at 4 °C to remove cell debris; tissue samples were homogenized in PBS containing protease inhibitors, then centrifuged at 12,000× *g* for 15 min at 4 °C, and the supernatants were collected. Subsequently, all clarified supernatants were aliquoted and stored at −80 °C for later use. Mouse ELISA kits (IL-17A, E-EL-M0047, Elabscience; IL-6, E-EL-M0044, Elabscience; TNF-α, E-EL-M3063, Elabscience) were used to measure the concentrations of IL-17A, IL-6, and TNF-α in the cell culture supernatants and tissue supernatants, respectively, strictly following the manufacturers’ instructions. Absorbance (OD) was measured at a wavelength of 450 nm, and concentrations were calculated based on the recombinant protein standard curves provided with each kit. All samples and standards were tested in triplicate to ensure statistical reliability.

### 2.22. Flow Cytometry Assay

T cells were activated using Dynabeads™ Mouse T Cell Activator CD3/CD28 (11456D, Gibco, Waltham, MA, USA), and IL-2 was supplemented to promote cell proliferation and long-term culture (212-12-20UG, Gibco). For Th17 and Treg polarization, the activated T cells were cultured under corresponding polarized induction conditions. Specifically, Treg polarization was induced by the addition of recombinant TGF-β (5 ng/mL) (7666-MB, R&D Systems, Minneapolis, MN, USA); Th17 polarization was induced by recombinant TGF-β combined with IL-6 (20 ng/mL) (E-AB-F1207A, Elabscience). After continuous polarized culture for 72 h, the cells were collected for subsequent phenotypic detection. To quantify the proportions of Th17 and Treg cells, flow cytometry was performed following standardized protocols. For surface marker staining, the single-cell suspensions were incubated with FITC-conjugated anti-mouse CD4 (E-AB-F1353C, Elabscience) and APC-conjugated anti-mouse CD25 (E-AB-F1102E, Elabscience) antibodies for 25 min at 4 °C in the dark.

For intracellular cytokine and transcription factor staining, cells were fixed and permeabilized using a commercial Fixation/Permeabilization Kit (00-5523-00, eBioscience, San Diego, CA, USA). Subsequently, the cells were incubated with APC-conjugated anti-mouse IL-17A (E-AB-F1199UE, Elabscience) or PE-conjugated anti-mouse Foxp3 (E-AB-F1238D, Elabscience) antibodies for 25 min at 4 °C in the dark. Data acquisition was performed using a custom-configured four-laser LSR II flow cytometer (BD Biosciences, Milpitas, CA, USA). The resulting data were analyzed using FlowJo software (v10.x; FlowJo LLC).

### 2.23. Western Blot Analysis

Total protein was extracted from the harvested skin tissues, in vitro-cultured T cells, and DPCs using RIPA lysis buffer (Solarbio, China) supplemented with protease and phosphatase inhibitors. Protein concentrations were quantified using a BCA Protein Assay Kit (PC0020, Solarbio, China) according to the manufacturer’s instructions.

Equivalent amounts of protein (30–50 μg) were separated via SDS-polyacrylamide gel electrophoresis (SDS-PAGE) and subsequently transferred onto polyvinylidene fluoride (PVDF) membranes (Millipore, Burlington, MA, USA). After blocking with 5% non-fat milk in TBST (Tris-buffered saline containing 0.1% Tween-20) for 1 h at room temperature, the membranes were incubated overnight at 4 °C with the following primary antibodies: anti-IL-17A (A12454, ABclonal, 1:3000), anti-NF-κB (80979-1-RR, Proteintech, Rosemont, IL, USA, 1:10,000), anti-p-NF-κB (AP1460, ABclonal, 1:10,000), anti-DKK1 (A2562, ABclonal, 1:700), anti-Wnt3A (DF6113, Affinity, Nanjing, China, 1:1000), anti-β-catenin (51067-2-AP, Proteintech, 1:10,000), and anti-GAPDH (81640-5-RR, Proteintech, 1:20,000) as an internal control.

The following day, the membranes were washed three times with TBST and incubated with HRP-conjugated secondary antibodies (SA00001-2, Proteintech, 1:20,000) for 1 h at room temperature. Protein bands were visualized using an enhanced chemiluminescence (ECL) kit (Thermo Fisher Scientific, USA). The immunoreactive band intensities were quantified via densitometry using ImageJ software, and the expression levels of the target proteins were normalized to GAPDH.

### 2.24. Immunofluorescence Staining

For immunofluorescence analysis, both processed skin tissues and in vitro-cultured cells were fixed with 4% paraformaldehyde for 20 min and permeabilized with 0.2% Triton X-100 for 15 min. After blocking with 5% bovine serum albumin (BSA) for 1 h at room temperature to minimize non-specific binding, the samples were incubated overnight at 4 °C with the specified primary antibodies (anti-Ki67 (1:200) A20018, ABclonal). The following day, the samples were washed three times with PBS and incubated with fluorophore-conjugated secondary antibodies for 1 h at room temperature in the dark. Cell nuclei were counterstained with DAPI (G1012, Servicebio) for 5 min. Fluorescence images were acquired using a laser-scanning confocal microscope (FV3000, Olympus, Tokyo, Japan).

### 2.25. Molecular Docking

Molecular docking experiments were conducted to evaluate the potential interactions between Plao-ELNs metabolites and the key IL-17A protein. Major metabolites (including Methyl deacetylasperulosidate, 5-O-(E)-p-coumaroylquinic acid, and Apigenin 7-glucoside) were obtained from the PubChem database, while the crystal structure of IL-17A was retrieved from the Protein Data Bank (PDB: 5HI4). Briefly, the receptor protein was prepared by removing water molecules, adding polar hydrogen atoms, and assigning Gasteiger partial charges. The docking search box was defined as follows: Center: center_x = 65.044, center_y = −23.031, center_z = −48.006; Grid box dimensions: size_x = 40 Å, size_y = 40 Å, size_z = 40 Å.

A blind docking strategy was used in this study. The relatively large dimension along the Z-axis was selected to cover a broad surface area of the IL-17A homodimer and to allow global screening of potential ligand-binding regions rather than restricting the search to a predefined binding pocket. Using the AutoDock Tools software (version 1.5.7), the protein and ligand structures were preprocessed. Docking calculations were performed using Auto-Dock Vina software, with the docking grid centered on the ligand-binding region (or active pocket) of IL-17A. The lowest-energy binding conformation for each protein-ligand complex was selected and further analyzed and visualized using PyMOL (version 3.0.3).

### 2.26. Statistical Analysis

All data are expressed as mean ± SD and were analyzed using GraphPad Prism 10.1.2. Differences between two groups were evaluated by the unpaired *t*-test, while multiple group comparisons were performed via one-way ANOVA followed by Bonferroni’s post hoc test. Each experiment was repeated at least three times (n ≥ 3). Statistical significance was defined as *p* < 0.05.

## 3. Results

### 3.1. Isolation and Characterization of Plao-ELNs

A schematic representation of the procedure for isolating Plao-ELNs from *P. orientalis* via differential ultracentrifugation is shown in Figure 1A. Morphological assessment by TEM revealed that, after negative staining, the isolated particles displayed the cup-shaped morphology commonly observed for extracellular vesicles (Figure 1B); this appearance is a known drying artifact of the negative-staining procedure and does not necessarily reflect the native vesicle geometry in solution. Low-magnification TEM fields further confirmed a homogeneous vesicle population (Figure S1). NTA was further applied to quantify the particle properties, showing a particle concentration of 7.7 × 10^11^ particles/mL and an average diameter of approximately 119.5 nm (Figure 1C). Figure 1D showed an average zeta potential of −10.65 mV for Plao-ELNs. To evaluate the protein profile, Plao-ELNs with a total protein concentration of 9.8 mg/mL were subjected to SDS-PAGE followed by Coomassie Brilliant Blue staining, which identified a distinct distribution of protein bands in Plao-ELNs (Figure 1E), thereby confirming the successful enrichment of vesicle-associated proteins. Furthermore, untargeted metabolomic analysis revealed that the primary cargo of Plao-ELNs comprised a diverse array of bioactive compounds, including flavonoids, terpenoids, phenylpropanoids, sugars, and glycosides (Figure 1F). Mapping these metabolites to predicted targets and intersecting the results with AGA-associated differentially expressed genes generated a metabolite-target-gene network (Figure 1G), nominating candidate axes for subsequent validation.

### 3.2. Synthesis and Characterization of HAMA/CP Composite Hydrogels

The synthesis of the HAMA/CP composite hydrogel was performed according to the protocol illustrated in Figure 2A. Under 365 nm UV irradiation, the HAMA/CP precursor solution underwent rapid photo-crosslinking within 30 s to form a stable, integrated hydrogel network (Figure 2B). Macroscopic evaluation demonstrated that the resulting composite hydrogel possessed robust adhesive properties, as evidenced by its ability to conformally adhere to a latex surface (Figure 2C), suggesting its potential for stable skin attachment.

FTIR spectroscopy was applied to verify the chemical structure and successful fabrication of the HAMA/CP hydrogel, and the entire FTIR spectra of HA/MA/CP and HAMA/CP were obtained (Figure 2D and Figure S2). Compared with the spectrum of native HA, the HAMA spectrum exhibited prominent new peaks at 1637 cm^−1^ (C=C stretching of the methacrylate group) and 1700–1710 cm^−1^ (ester C=O stretching), confirming that methacrylate groups were successfully grafted onto the HA backbone [26]. This confirms that methacrylate groups were successfully grafted onto the HA backbone. Following the introduction of CP, a strong and broad hydroxyl (-OH) stretching absorption peak was observed at approximately 3400 cm^−1^, and a C-H stretching peak was observed near 2900 cm^−1^, while the characteristic features of the CP backbone were reflected in the C-O-C glycosidic bond and C-OH stretching vibrations in the 1000–1150 cm^−1^ range [27]. These results demonstrated that CP was successfully incorporated into the HAMA network without disrupting the fundamental hydrogel structure. SEM characterization further revealed that the lyophilized HAMA/CP hydrogel displayed a highly porous and interconnected microarchitecture (Figure 2E), which facilitates nutrient exchange and sustained release of Plao-ELNs.

Hemocompatibility, antibacterial activity, and cell migration-promoting capacity were further evaluated to verify the biosafety and functional performance of the composite hydrogel. The in vitro hemolysis assay confirmed negligible hemolytic activity of the HAMA/CP hydrogel, indicating excellent hemocompatibility and satisfactory biosafety for biomedical applications (Figure 2F). Notably, the HAMA/CP hydrogel exhibited observable antibacterial phenotype against both *E. coli* and *S. aureus*, whereas the pure HAMA hydrogel showed negligible antibacterial effects (Figure 2G). This enhanced antibacterial performance is likely attributable to the intrinsic antibacterial properties of the incorporated cactus polysaccharides. Furthermore, the effect of the hydrogel on the regenerative capacity of DPCs was evaluated via an in vitro scratch assay. The results demonstrated that the HAMA/CP hydrogel-treated group achieved a significantly higher wound closure rate compared to both the control and HAMA groups at specific time points post-scratch (Figure 2H,I).

### 3.3. Fabrication and Characterization of P-MN

Building on the HAMA/CP composite hydrogel characterized above, we developed a dissolvable hydrogel microneedle system to encapsulate Plao-ELNs (P-MN) for intradermal delivery (Figure 3A). A HAMA/CP precursor solution containing Plao-ELNs was cast into a PDMS mold and subjected to UV-initiated photo-crosslinking for 30 s. The resulting dried MNs were then carefully demolded. Representative optical microscopy image (Figure 3B) and SEM (Figure 3C) confirmed that P-MN exhibited a well-defined conical morphology with sharp tips.

The dissolution kinetics of P-MN was evaluated by immersion in PBS. P-MN dissolved rapidly upon immersion in PBS, with 50% of the needle mass lost within 1 min (Figure 3D). Plao-ELNs were recovered from dissolved P-MN and examined by TEM. The released Plao-ELNs retained their characteristic vesicular morphology, with intact membrane structures and no obvious membrane disruption or extensive aggregation (Figure S3). To verify the distribution of the therapeutic payloads, Plao-ELNs were labeled with DiI (red), whereas the needle body was visualized by physically entrapped DAPI (blue), which served only to demarcate the needle outline rather than to covalently label the HAMA/CP matrix. Fluorescence microscopy images (Figure 3E) revealed that Plao-ELNs were uniformly encapsulated and concentrated within the microneedle tips. The skin penetration capacity of P-MN was further evaluated on mouse dorsal skin. The MNs successfully bypassed the stratum corneum, creating distinct and uniform microchannels corresponding to the needle array (Figure 3F). H&E staining of the puncture sites further confirmed the effective penetration of the epidermis, with conical cavities extending into the superficial dermis (Figure 3G).

Systemic tolerability was assessed by histopathology of major organs (heart, liver, spleen, lung, kidney) at the end of treatment; no necrosis, inflammatory infiltration, or fibrosis was detected in P-MN group (Figure S4). Finally, the intradermal retention and permeation of P-MN were monitored using an in vivo imaging system (IVIS). Compared with the PBS group, the P-MN group exhibited significantly higher fluorescence intensity and a prolonged retention time (Figure 3H,I).

### 3.4. Evaluation of the Therapeutic Efficacy of P-MN in an AGA Mouse Model

To evaluate the hair regrowth efficacy of P-MN, a testosterone-induced AGA model was established in C57BL/6 mice (Figure 4A). This model recapitulated the key pathological features of androgen-driven hair loss, including delayed anagen entry and follicular miniaturization [28,29]. Topical MXD was set as the positive control to provide a clinical therapeutic benchmark. Dorsal skin pigmentation and hair regrowth were continuously monitored throughout the 28-day treatment period.

The AGA group exhibited persistent alopecia with negligible melanin deposition until day 17, confirming the successful induction of androgen-mediated hair loss. In sharp contrast, melanin deposition—a precursor to anagen entry—appeared as early as day 10 in the Control, MXD, and P-MN groups. In the B-MN group, the mean onset of pigmentation was approximately day 10–11, although individual animals showed considerable variability and the latest animal did not develop visible pigmentation until approximately day 17 (Figure 4B,D). By day 28, hair coverage in the P-MN group was higher than that in the MXD group and closely approached the density of the Control group (Figure 4C). Consistent with macroscopic observations, SEM analysis further verified that the hair shaft diameters in the P-MN and MXD groups were significantly thicker than those in the AGA and B-MN groups (Figure 4E,F).

H&E histological staining was performed to further assess hair follicle structural restoration. The AGA group exhibited typical telogen follicle characteristics, including follicular atrophy, decreased follicle number, and upward migration of hair bulbs toward the superficial dermis. Notably, P-MN treatment significantly reversed these pathological alterations. The P-MN group exhibited a robust increase in hair follicle density and the repositioning of the hair bulbs into the deep dermis, accompanied by enlarged dermal papillae and a predominant population of follicles in the anagen phase (Figure 4G,H).

### 3.5. Mechanistic Insights into P-MN Mediated Hair Regrowth

Transcriptomic profiling was performed on dorsal skin tissues from the AGA and P-MN groups. Differential expression analysis yielded a total of 24,522 genes, among which 35 genes were significantly upregulated and 185 genes were downregulated following P-MN treatment. Global transcriptional alterations were visualized via a volcano plot (Figure 5A) and a hierarchical clustering heatmap (Figure 5B). Importantly, GO and KEGG enrichment analyses highlighted that the DEGs were predominantly enriched in immune-regulatory pathways and the IL-17 signaling pathway (Figure 5C,D), indicating a critical immunomodulatory function of Plao-ELNs. The DEGs obtained from transcriptomic screening were imported into the STRING database (Version: 12.5) to construct a protein–protein interaction (PPI) network. After defining species and confidence thresholds, isolated nodes were removed to optimize the network. The refined PPI network was subsequently imported into Cytoscape (Version: 3.10.0). The CytoHubba plugin and multiple topological algorithms were applied to score and rank hub genes. Core genes were screened according to degree, betweenness centrality, and closeness centrality rankings. Ultimately, IL-17A was identified as the key gene driving P-MN-mediated hair regeneration (Figure 5E).

To evaluate the binding capacity of major Plao-ELN metabolites to IL-17A, metabolite composition was analyzed via LC-MS/MS. Plao-ELNs were found to be enriched in diverse bioactive components. A total of 43 major metabolites were identified in positive ion mode, including Methyl deacetylasperulosidate and 5-O-(E)-p-coumaroylquinic acid, while 35 major metabolites were detected in negative ion mode, including Apigenin 7-glucoside (Figure 5F and Table S1). Among the candidates, 5-O-(E)-p-coumaroylquinic acid exhibited the most favorable predicted binding affinity, with a docking score of −9.7 kcal/mol, followed by Apigenin 7-glucoside (−9.4 kcal/mol) and Methyl deacetylasperulosidate (−8.2 kcal/mol) (Figure 5G). Structural analysis of the representative binding conformations revealed that these metabolites preferentially interacted with residues within the hydrophobic groove at the IL-17A homodimer interface (PDB: 5HI4) (Figure 5H).

### 3.6. Mechanisms of Action of P-MN in Regulating the Immune Microenvironment of Hair Follicles for the Treatment of AGA

The infiltration of Th17 and Treg cells in skin tissues was analyzed. Compared with the Control group, AGA mice exhibited significantly increased Th17 cell infiltration and reduced Treg cell abundance, resulting in an elevated Th17/Treg ratio (Figure 6A–D). P-MN treatment markedly reversed these alterations, indicating restoration of immune homeostasis within the follicular microenvironment.

Given the critical role of Th17 cells in IL-17A-mediated inflammation responses, the activation status of the IL-17A/NF-κB signaling pathway was further evaluated. As shown in Figure 6E–G, the protein expression levels of IL-17A and NF-κB (p-NF-κB) were significantly increased in AGA skin tissues compared with those in the Control group. Consistently, ELISA analysis revealed elevated levels of IL-17A and the downstream pro-inflammatory cytokines IL-6 and TNF-α. In contrast, P-MN treatment significantly reduced the expression of IL-17A and NF-κB (p-NF-κB), accompanied by decreased production of IL-17A, IL-6, and TNF-α (Figure 6H–J).

### 3.7. Downstream Mechanisms of P-MN in Releasing Wnt/β-Catenin Suppression and Promoting Hair Follicle Proliferation

We investigated the crosstalk between the resolved inflammatory niche and the canonical hair growth signaling pathways. DKK1, a potent and well-established antagonist of the Wnt/β-catenin pathway, is known to be transcriptionally upregulated by NF-κB-driven inflammatory cascades. Western blot analysis revealed that DKK1 expression was markedly elevated in the skin tissues of the AGA group, demonstrating that the abnormal activation of the IL-17A/NF-κB axis resulting from AGA immune imbalance directly drives the pathological upregulation of this Wnt inhibitor (Figure 7A,B). In contrast, P-MN treatment significantly downregulated DKK1 expression, thereby effectively relieving its antagonistic pressure on the Wnt/β-catenin signaling axis (Figure 7A,C,D).

Ki67 immunofluorescence staining was performed on day 14 after treatment. As shown in Figure 7E,F, the fluorescence intensity of Ki67 in the hair bulb matrix was markedly reduced in the AGA group, while P-MN treatment significantly restored Ki67 expression levels. In addition, the P-MN group exhibited higher Ki67 signals than the B-MN group, with proliferative activity close to that of the Control group.

### 3.8. Plao-ELNs Regulate CD4^+^ T Cell Differentiation In Vitro

Naïve CD4^+^ T cells were cultured under Treg-or Th17-polarizing conditions in the presence or absence of Plao-ELNs. Plao-ELNs were added at a final protein concentration of 100 µg/mL, equivalent to 7.86 × 10^9^ particles/mL (based on a stock concentration of 7.7 × 10^11^ particles/mL) (Figure 8A). Flow cytometric analysis showed that Plao-ELNs significantly increased the proportion of Treg cells under Treg-inducing conditions, whereas the frequency of Th17 cells was markedly reduced under Th17-polarizing conditions (Figure 8B–E).

Western blot analysis demonstrated that Plao-ELNs significantly reduced the expression of IL-17A and NF-κB (p-NF-κB) (Figure 8F–H). Consistently, ELISA resulted revealed decreased secretion of IL-17A, IL-6, and TNF-α in the culture supernatants following Plao-ELNs treatment (Figure 8I–K).

### 3.9. Plao-ELNs Modulate the In Vitro Immune Microenvironment to Promote DPCs Proliferation via the DKK1 Pathway

Primary DPCs were first isolated and characterized. ALP staining revealed strong positive signals in the cytoplasm, indicating that DPCs retained their characteristic inducer phenotype. Furthermore, immunofluorescence staining showed significant β-catenin expression along the cell membrane, consistent with the established phenotypic characteristics of DPCs (Figure 9A).

DiI-labeled Plao-ELNs were co-cultured with DPCs and observed using a confocal laser scanning microscope. As shown in Figure 9B, strong red fluorescence was distributed in the perinuclear cytoplasm, indicating the efficient internalization of Plao-ELNs by DPCs. According to the experimental workflow illustrated in Figure 9C, conditioned medium (CM) was harvested from Th17-polarized CD4^+^ T cells with or without Plao-ELNs treatment and then applied to DPCs for further intervention.

Western blot analysis showed that DPCs cultured with CM from Plao-ELN-treated Th17 cells exhibited significantly lower DKK1 expression than those cultured with untreated Th17 cell supernatant. Correspondingly, the downstream Wnt/β-catenin signaling activity was markedly enhanced in the Plao-ELNs-CM group (Figure 9D–G). Furthermore, Ki67 immunofluorescence staining revealed enhanced proliferative activity in DPCs treated with Plao-ELNs-CM, as evidenced by significantly higher Ki67 fluorescence intensity compared to the group treated with untreated medium (Figure 9H,I).

## 4. Discussion

AGA pathogenesis is dominated by progressive hair follicle miniaturization driven by dysregulated hair cycle progression, including an abbreviated anagen stage and persistent telogen retention [30]. Such pathological alterations gradually transform thick terminal hairs into fine vellus hairs, ultimately causing progressive hair thinning [31]. Clinical treatment of AGA remains limited by the limited efficacy of existing therapies in improving the deteriorating microenvironment of hair follicles [32]. Against this backdrop, PELNs have gradually emerged as a powerful tissue repair platform due to their versatility in modulating the microenvironment [33]. The present study demonstrated that local delivery of Plao-ELNs via hydrogel microneedles effectively reverses hair follicle miniaturization and facilitates the transition of hair follicles from the telogen to the anagen phase. Importantly, we elucidated a previously unreported mechanism by which Plao-ELNs mediate follicular immune microenvironment remodeling. These findings are highly consistent with the existing literature on plant extracts and plant-derived extracellular nanovesicles, which demonstrate that these components can promote hair regrowth by inducing the anagen phase, increasing hair follicle size and density, and restoring normal hair follicle morphology [34,35].

AGA pathogenesis is no longer considered a simple androgen-dependent disorder restricted to follicular epithelial cells. Instead, the disruption of follicular immune microenvironment homeostasis—particularly the loss of follicular immune privilege—is increasingly acknowledged as a pivotal driver of follicle miniaturization and premature anagen arrest [36]. Under normal physiological conditions, the hair follicle maintains relative immune privilege, with Treg cells preserving a localized immunosuppressive niche that protects hair follicle cells and their progeny from autoimmune attack [37]. In the pathological state of AGA, however, a marked Th17/Treg imbalance occurs, characterized by excessive expansion of pro-inflammatory Th17 cells and decreased abundance of protective Treg cells. This immune shift promotes excessive secretion of IL-17A and further activates the downstream pro-inflammatory NF-κB signaling cascade [38,39]. The persistent perifollicular inflammatory microenvironment not only causes local tissue inflammatory damage but also severely impairs the physiological function of DPCs. Notably, the present study revealed a critical crosstalk between Th17/Treg immune homeostasis and the intracellular DKK1/Wnt/β-catenin signaling axis in DPCs. Local delivery of Plao-ELNs via hydrogel microneedles effectively remodels follicular T-cell distribution and corrects the pathological Th17/Treg imbalance. Restoration of immune homeostasis inhibits inflammation-induced DKK1 upregulation in DPCs, relieves DKK1-mediated suppression of canonical Wnt/β-catenin signaling, and ultimately facilitates DPCs proliferation and hair follicle regeneration. By reshaping the inflammatory and immunosuppressive perifollicular niche, Plao-ELNs appear to shift a regeneration-inhibitory microenvironment into a pro-regenerative state, thereby promoting hair follicle recovery and hair regrowth.

*P. orientalis* has long been used in traditional medicine for alopecia treatment with well-documented ethnopharmacological value. Previous studies on its hair-regenerative effects have mainly focused on bioactive phytochemicals in crude extracts, especially flavonoid monomers such as quercetin. These components promote hair growth by improving perifollicular microcirculation, alleviating oxidative stress, and enhancing the proliferation and biological activity of DPCs [40,41,42]. While such studies have convincingly demonstrated the pharmacological efficacy of *P. orientalis*, the underlying mechanisms have largely been interpreted within the framework of direct actions of exogenous small molecules on the follicular vasculature or terminal effector cells. Consequently, little attention has been paid to the potential role of *P. orientalis*-derived bioactive agents in regulating the follicular immune microenvironment, particularly the adaptive immune network that critically governs hair follicle homeostasis and regeneration. In contrast to conventional crude extracts or isolated phytochemicals, the Plao-ELNs developed in the present study represent a fundamentally distinct therapeutic modality. By preserving the natural lipid bilayer architecture and encapsulating a diverse repertoire of bioactive cargos characteristic of plant-derived extracellular nanovesicles, Plao-ELNs function not merely as a source of therapeutic molecules but as a sophisticated biogenic delivery platform capable of mediating intercellular communication. This unique biological property enables Plao-ELNs to intervene at the level of immune regulation, thereby extending the therapeutic paradigm of Plao from traditional concepts of “microcirculation enhancement” and “nutritional support” toward precise modulation of the follicular immune niche and restoration of immune privilege.

Recently, Shang et al. demonstrated that PO-EVs ameliorate AGA through a multifaceted mechanism involving hair follicle activation, microenvironmental restoration, and angiogenesis enhancement. Consistent with these findings, our results further validate the therapeutic potential of Plao-ELNs in AGA treatment. Notably, we further delineated the molecular cascade linking immune regulation to hair follicle regeneration through the IL-17A/NF-κB/DKK1 signaling axis. DKK1 is a well-established antagonist of Wnt/β-catenin signaling and plays a pivotal role in promoting hair follicle miniaturization and premature entry into the catagen phase [43,44]. Notably, Plao-ELNs do not exert non-specific anti-inflammatory effects. Instead, they specifically block the pathological IL-17A-NF-κB signaling cascade initiated by dysregulated T-cell responses, thereby preventing the transcriptional upregulation of DKK1 [45]. The consequent release of Wnt/β-catenin signaling from DKK1-mediated inhibition reactivates regenerative programs within dermal papilla cells and provides the molecular cues necessary for hair follicles to re-enter the anagen phase. Collectively, these results establish a mechanistic framework whereby Plao-ELNs coordinate immune homeostasis and follicular regeneration, highlighting the IL-17A/NF-κB/DKK1/Wnt/β-catenin axis as a previously underappreciated therapeutic target for AGA intervention.

In addition to the intrinsic therapeutic activity of Plao-ELNs, the design of an appropriate biomaterial carrier is crucial for maximizing their regenerative efficacy. Extracellular vesicle-based therapeutics often face challenges associated with rapid diffusion, limited local retention, and premature degradation following administration, which may substantially restrict their biological activity in vivo [46]. To address these limitations, we developed a multifunctional HAMA/CP composite hydrogel that served not only as a structural matrix for microneedle fabrication but also as a bioactive microenvironment capable of supporting hair follicle regeneration [47]. The excellent hemocompatibility and absence of detectable histopathological abnormalities in major organs demonstrated the favorable biosafety profile of the HAMA/CP platform, supporting its suitability for translational applications in alopecia treatment.

Notably, the incorporation of CP endowed the HAMA hydrogel with multifunctional bioactivity beyond its structural role. Consistent with previous reports describing the antimicrobial and tissue-repair-promoting properties of plant-derived polysaccharides [48], the HAMA/CP hydrogel exhibited an observable antibacterial phenotype against both *E. coli* and *S. aureus* while significantly enhancing DPCs migration, suggesting that CP actively contributes to the establishment of a pro-regenerative follicular microenvironment through the regulation of microbial homeostasis and cellular behavior. In addition, the highly porous and interconnected architecture of the HAMA/CP matrix provides abundant loading sites and diffusion channels for Plao-ELNs, enabling the hydrogel to function as an effective local reservoir for sustained nanovesicle delivery. Correspondingly, in vivo imaging demonstrated prolonged intradermal retention and enhanced accumulation of Plao-ELNs following administration of the hydrogel microneedle system. Such prolonged intradermal retention are particularly advantageous for extracellular vesicle-based therapies, as they prolong local bioavailability and facilitate continuous modulation of the follicular immune niche. Therefore, the HAMA/CP hydrogel not only creates a favorable microenvironment for hair follicle regeneration through its intrinsic antibacterial and pro-migratory activities but also amplifies the immunoregulatory and regenerative effects of Plao-ELNs by protecting them from rapid clearance and extending their therapeutic window.

Nevertheless, several limitations of this study should be acknowledged. First, molecular docking only provides in silico prediction, and further biochemical interaction experiments are required to verify the direct physical binding between Plao-ELN-derived bioactive metabolites and IL-17A. Second, the androgen-induced mouse AGA model cannot fully recapitulate the complex pathological features of human androgenetic alopecia scalp. Third, since Plao-ELNs are a mixture of multiple bioactive components, the exact contribution of each individual metabolite remains unclear and awaits future deconvolution studies. Fourth, RNA-seq was performed only on the AGA and P-MN groups; the B-MN and MXD groups were not sequenced, and mechanistic assays did not include all treatment groups, which limits the ability to fully separate the contribution of the Plao-ELNs cargo from that of microneedle application or the CP matrix alone. Fifth, the in vitro cumulative release kinetics of Plao-ELNs could not be established, because the needle matrix dissolved too rapidly in PBS; the conclusion of prolonged local delivery therefore rests on in vivo retention imaging rather than quantitative release profiles.

## 5. Conclusions

In conclusion, the therapeutic efficacy of Plao-ELNs in promoting hair follicle regeneration is mediated by their ability to modulate the follicular immune microenvironment, specifically through suppression of aberrant T-cell activation and inhibition of the IL-17A/NF-κB/DKK1 signaling cascade, thereby reactivating Wnt/β-catenin-driven regenerative pathways. Importantly, this immunoregulatory effect is significantly enhanced by the multifunctional HAMA/CP hydrogel platform, which not only serves as a sustained-release reservoir for Plao-ELNs but also provides structural support, antimicrobial protection, and promotion of dermal papilla cell migration. The synergistic interplay between the bioactive Plao-ELNs and the HAMA/CP matrix enables simultaneous regulation of immune, microbial, and regenerative components within the follicular niche, creating a favorable microenvironment that maximizes hair follicle regeneration. Collectively, these findings establish a mechanistic and materials-based framework for hair restoration therapy and highlight the translational potential of combining plant-derived extracellular vesicles with functional hydrogel delivery systems.

## Figures and Tables

**Figure 1:**
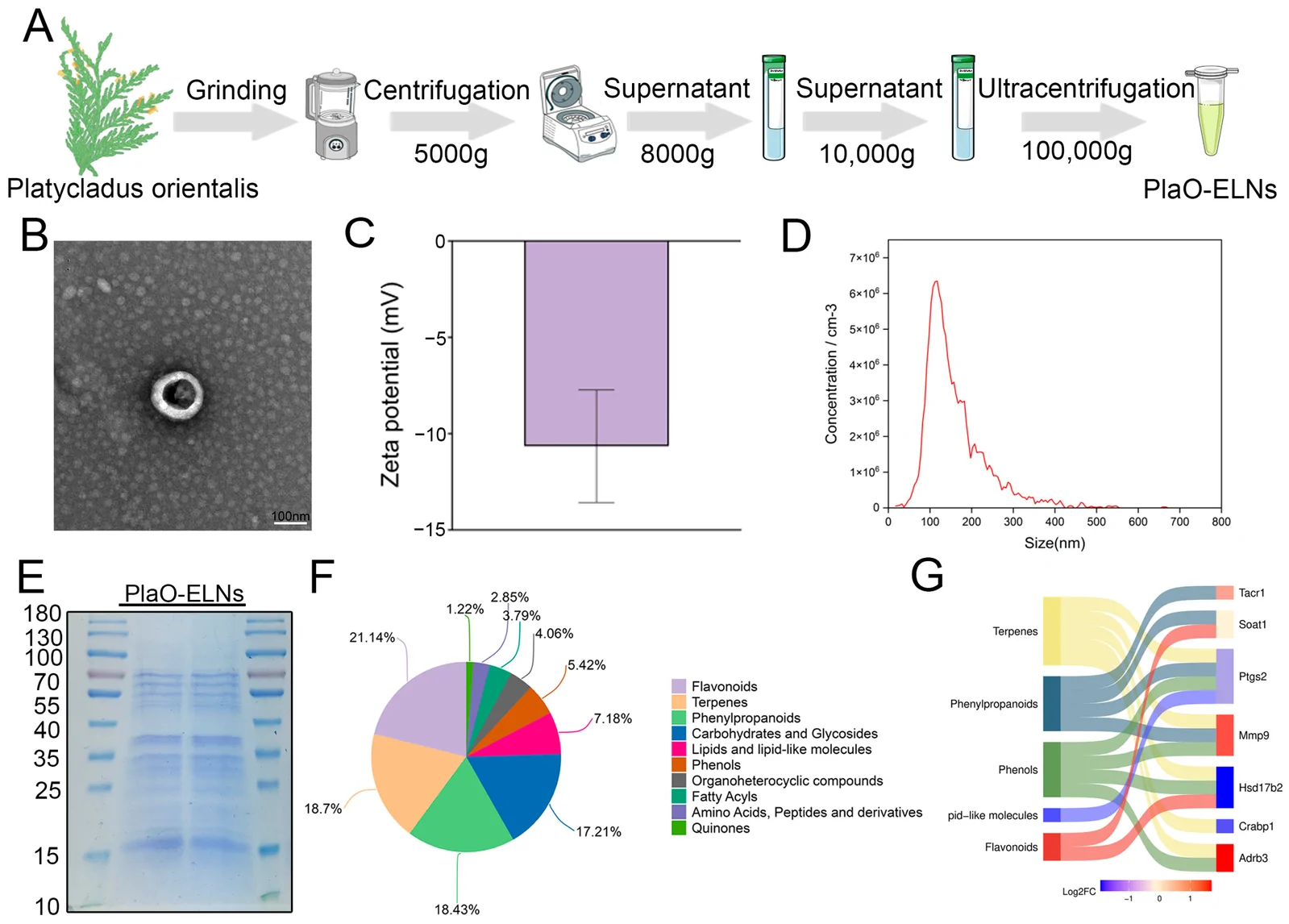
Isolation and characterization of Plao-ELNs. (**A**) Schematic illustration of the isolation procedure for Plao-ELNs. (**B**) Transmission electron microscopy (TEM) images showing the morphology of Plao-ELNs. (**C**) Zeta potential of Plao-ELNs. (**D**) Particle size distribution of Plao-ELNs. (**E**) Isolation and Coomassie Brilliant Blue staining analysis of proteins derived from Plao-ELNs. (**F**) Classification and relative abundance of major bioactive components identified in Plao-ELNs. (**G**) Sankey diagram depicting the relationships between the active constituents of Plao-ELNs, their predicted target genes, and AGA-related genes.

**Figure 2:**
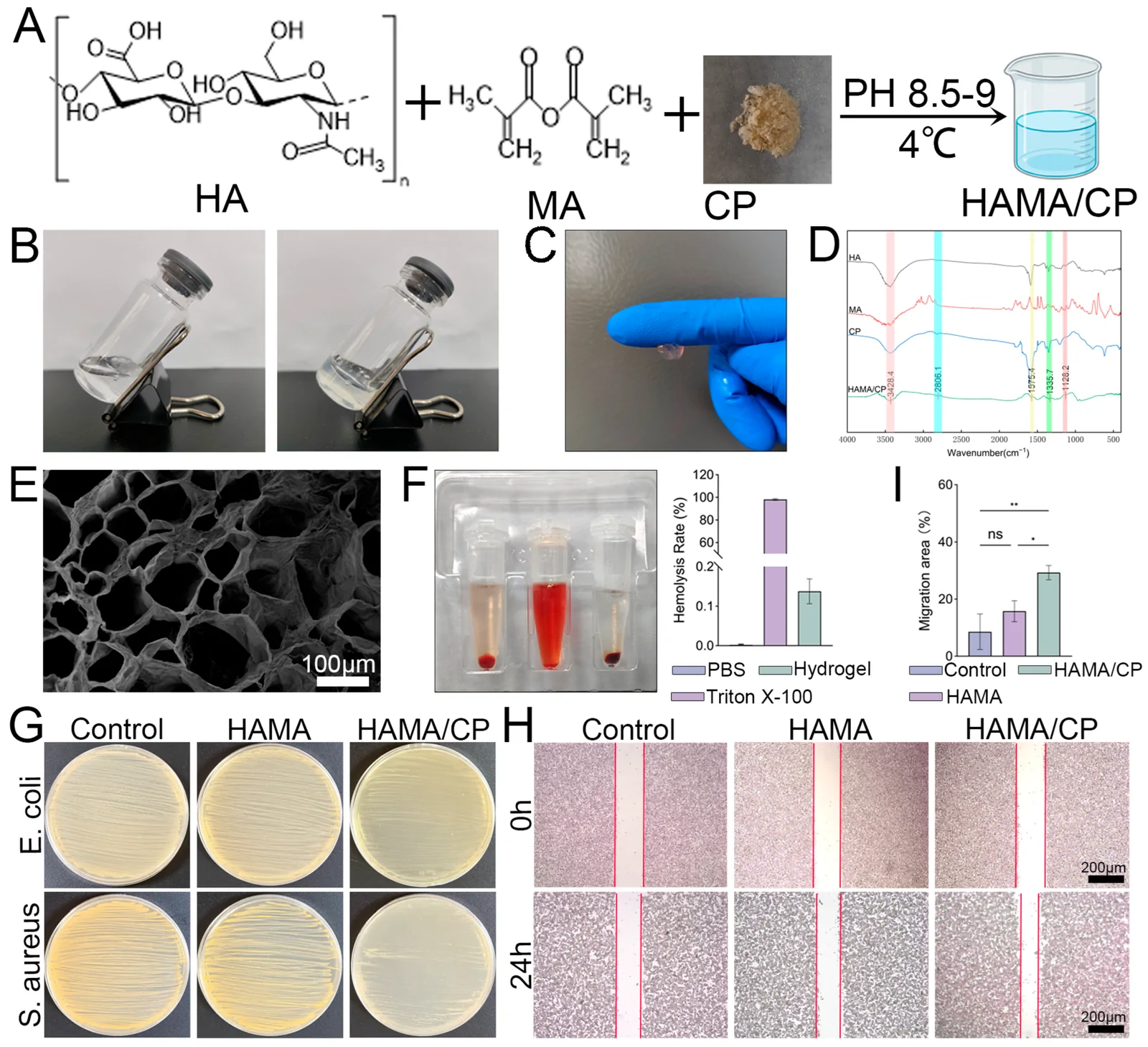
Synthesis and characterization of HAMA/CP composite hydrogels. (**A**) Schematic illustration of the fabrication process of HAMA/CP composite hydrogels. (**B**) Representative photographs of the HAMA/CP composite hydrogels. (**C**) Evaluation of the adhesive properties of HAMA/CP composite hydrogels. (**D**) FTIR spectra of HA, MA, CP, and HAMA/CP composite hydrogels. (**E**) SEM images showing the microstructure of HAMA/CP composite hydrogels. (**F**) Hemocompatibility assessment of HAMA/CP composite hydrogels by hemolysis assay. (**G**) Antibacterial activity of HAMA/CP composite hydrogels against *E. coli* and *S. aureus*. (**H**) Representative images of DPCs migration assessed by scratch wound-healing assay. (**I**) Quantitative analysis of DPC migration rates in different treatment groups. Data are presented as the mean ± SD (n = 3). ns, not significant; * *p* < 0.05; ** *p* < 0.01.

**Figure 3:**
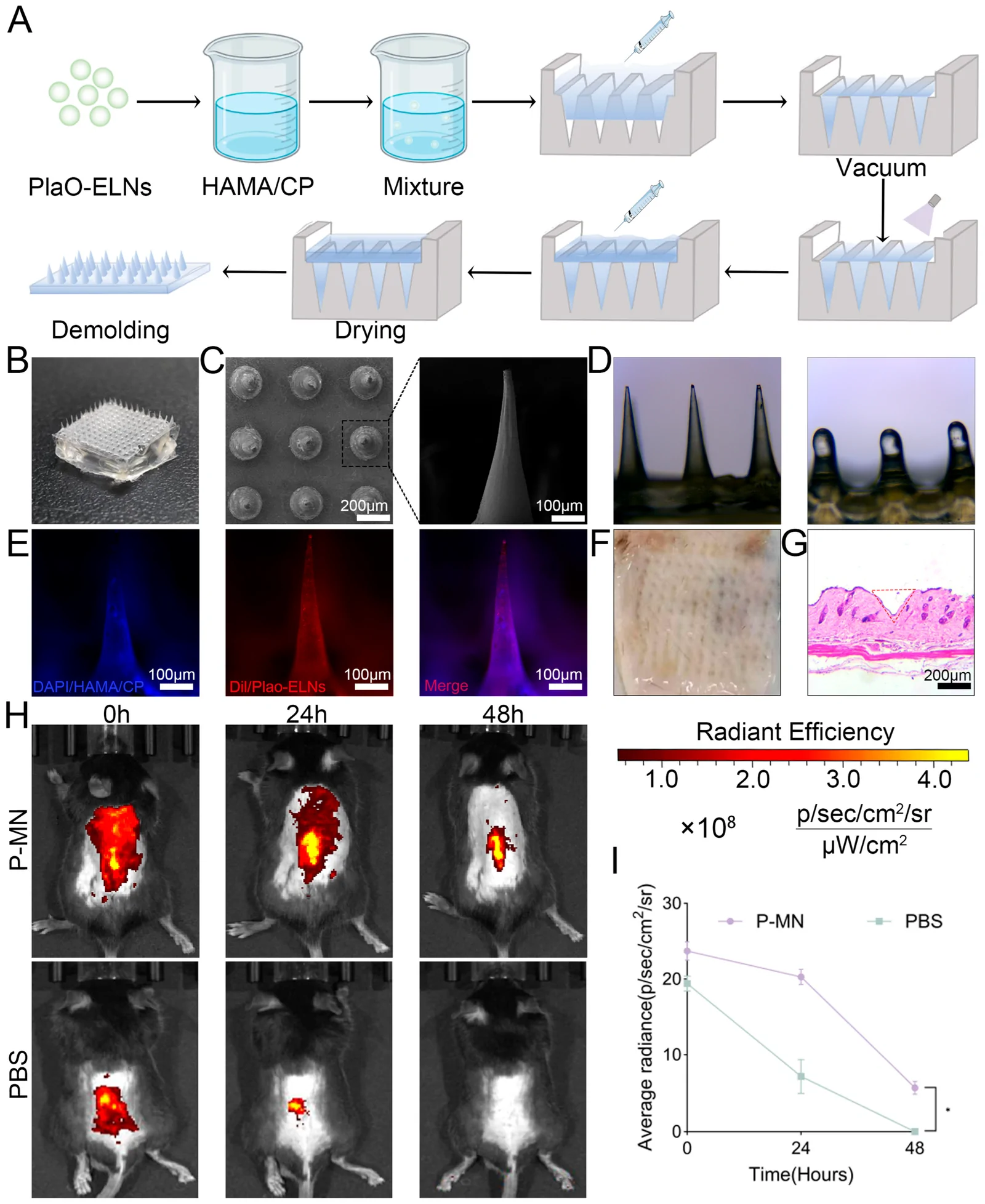
Fabrication and characterization of Plao-ELN-loaded microneedles. (**A**) Schematic illustration of the fabrication process of P-MN. (**B**) Representative optical microscopy image of the fabricated P-MN patch. (**C**) SEM image showing the morphology of P-MN. (**D**) In vitro degradation profile of the P-MN needle matrix. (**E**) Fluorescence microscopy image showing the distribution of Plao-ELNs within the P-MN structure. Plao-ELNs were labeled with DiI (red), and DAPI (blue) was physically entrapped in the matrix solely to demarcate the needle outline and does not covalently label HAMA/CP. (**F**) Representative image of microchannels formed in ex vivo mouse dorsal skin after P-MN insertion. (**G**) H&E staining of skin sections following P-MN insertion. (**H**,**I**) Representative in vivo fluorescence images and corresponding quantitative analysis of fluorescence retention in the dorsal skin following administration of DiI-labeled P-MN or PBS. Data are presented as the mean ± SD (n = 3 mice per group). * *p* < 0.05.

**Figure 4:**
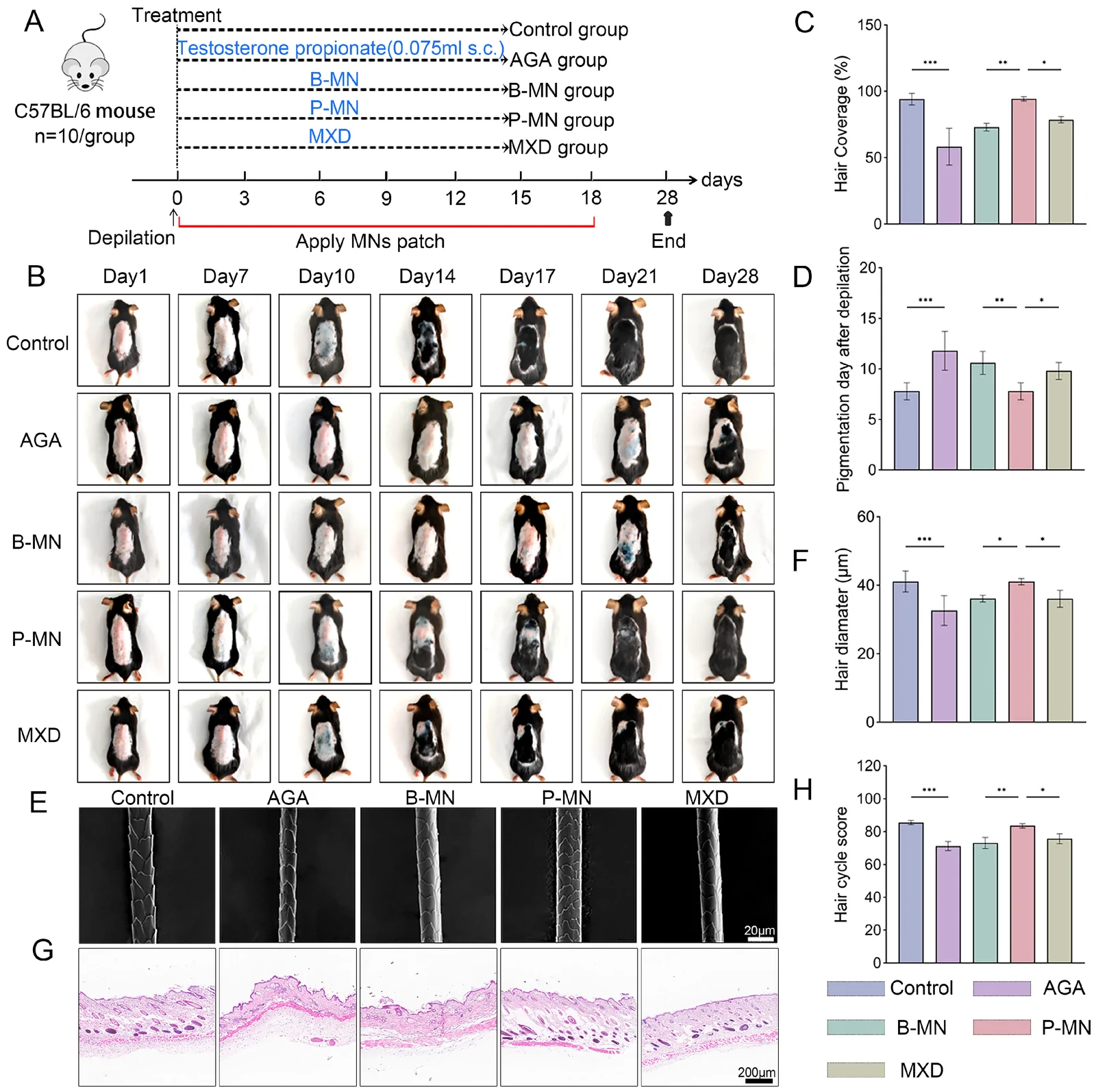
Evaluation of the therapeutic efficacy of P-MN in a mouse model of AGA. (**A**) Schematic illustration of AGA model establishment and treatment protocols. (**B**) Representative photographs showing dorsal hair regrowth in different treatment groups (n = 10 mice per group). (**C**) Quantitative analysis of dorsal hair coverage area in each group (n = 10 mice per group). (**D**) Quantitative analysis of the time to pigmentation onset in the dorsal skin of mice (n = 10 mice per group). (**E**) SEM images of regenerated hair shafts from different treatment groups (n = 3 mice per group, with ≥50 shafts measured per group). (**F**) Quantitative analysis of regenerated hair shaft diameter in each group (n = 3 mice per group, with ≥50 shafts measured per group). (**G**) H&E staining of dorsal skin sections following treatment (n = 3 independent skin samples from three different mice). (**H**) Quantitative analysis of hair cycle scores in different groups (n = 3 independent skin samples from three different mice). Data are presented as the mean ± SD. * *p* < 0.05; ** *p* < 0.01; *** *p* < 0.001 AGA compared to the Control group; P-MN compared to the B-MN group; P-MN compared to the MXD group.

**Figure 5:**
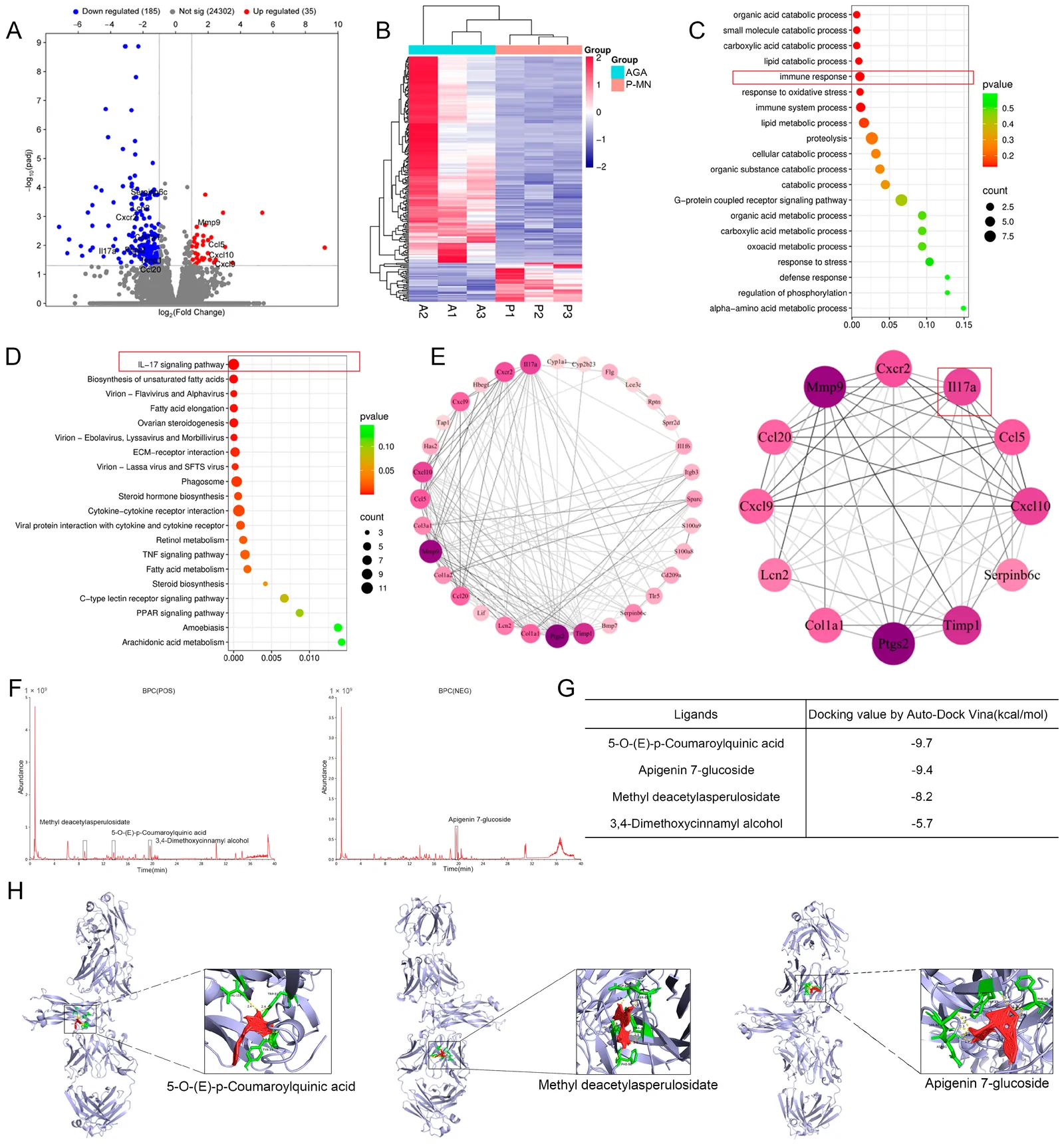
Transcriptomic analysis reveals the molecular mechanisms underlying P-MN-mediated hair regeneration. (**A**) Volcano plot showing differentially expressed genes (DEGs) between the AGA and P-MN groups. (**B**) Heatmap of DEGs between the AGA and P-MN groups. (**C**) GO enrichment analysis of DEGs. (**D**) KEGG pathway enrichment analysis of DEGs. Red squares mark the enriched immune-regulatory pathways and IL-17 signaling pathway. (**E**) PPI network of candidate genes constructed using Cytoscape. Different shades of pink represent distinct gene modules in the PPI network. (**F**) Chemical characterization of Plao-ELNs using LC-MS/MS in positive and negative ion modes. (**G**) Molecular docking of bioactive compounds from major Plao-ELNs with IL-17A. (**H**) Predicted binding sites of the Plao-ELNs compounds within the IL-17A homodimer interface. Different color indicate distinct binding pockets at the IL-17A homodimer interface.

**Figure 6:**
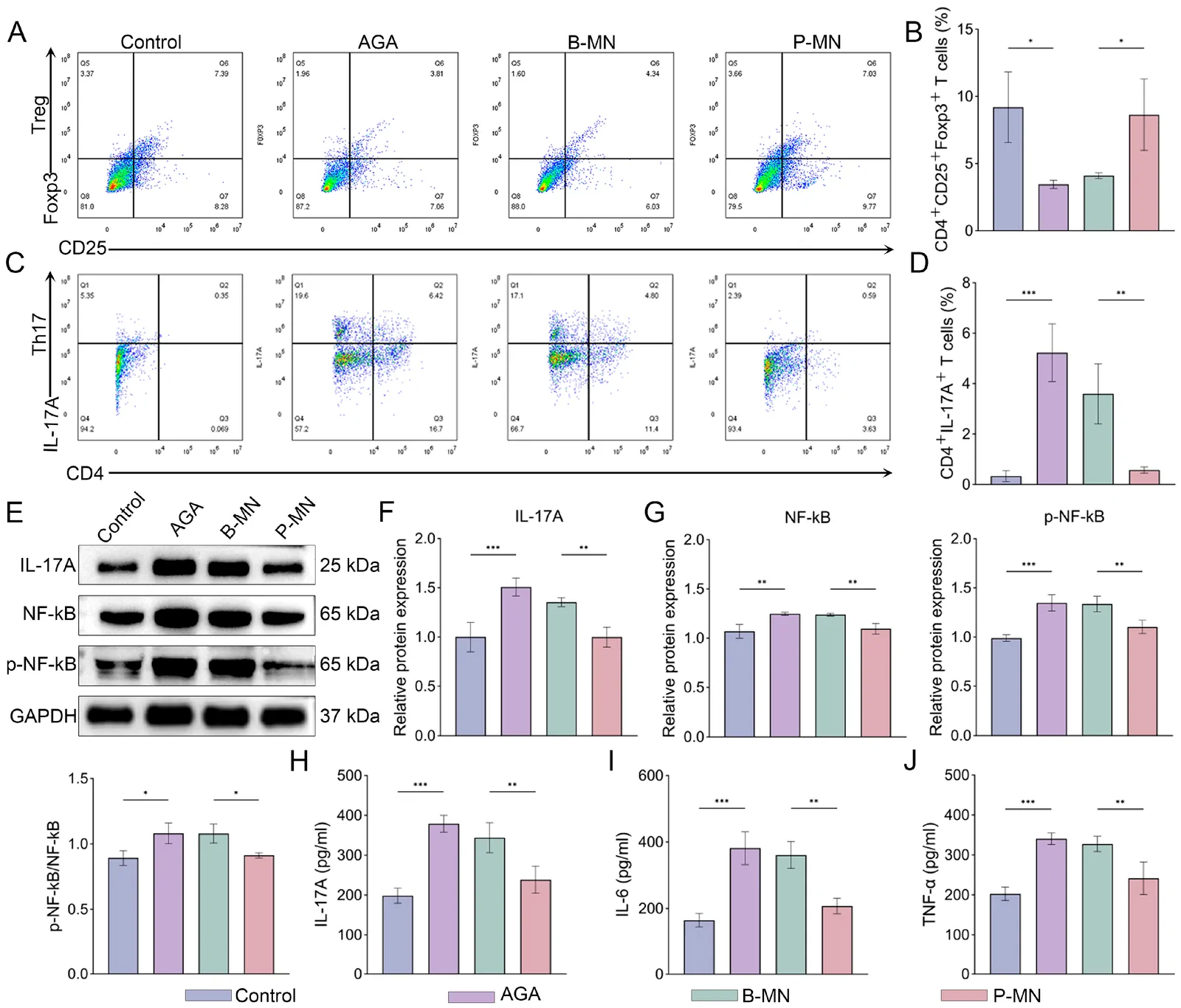
P-MN remodels the follicular immune microenvironment to ameliorate androgenetic alopecia. (**A**–**D**) Flow cytometric analysis and quantitative assessment of the proportions of Treg and Th17 cells in different treatment groups. (**E**) Western blot analysis of IL-17A, NF-κB, and phosphorylated NF-κB (p-NF-κB) protein expression in skin tissues. (**F**,**G**) Quantitative analysis of IL-17A, NF-κB, and p-NF-κB protein levels. (**H**–**J**) ELISA analysis of IL-17A, IL-6, and TNF-α levels in skin tissue samples from different groups. Data are presented as the mean ± SD (n = 3 independent skin samples from three different mice). * *p* < 0.05; ** *p* < 0.01; *** *p* < 0.001 AGA compared to the Control group; P-MN compared to the B-MN group.

**Figure 7:**
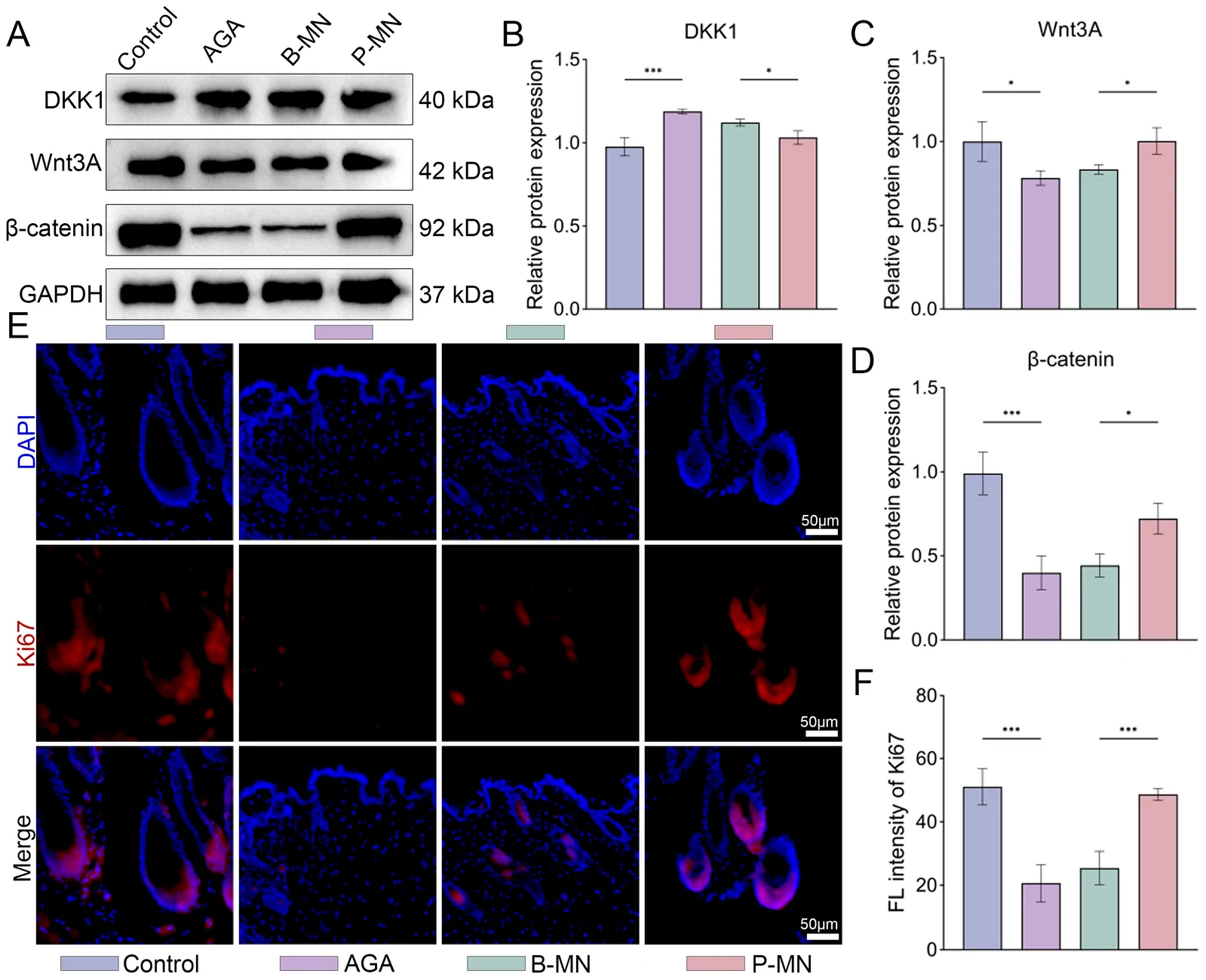
P-MN promotes hair follicle regeneration by alleviating DKK1-mediated suppression of Wnt/β-catenin signaling. (**A**) Western blot analysis of DKK1, Wnt3A, and β-catenin protein expression in skin tissues from different treatment groups. (**B**–**D**) Quantitative analysis of DKK1, Wnt3A, and β-catenin protein levels. (**E**) Representative immunofluorescence images of Ki67 expression in skin tissues from different groups. (**F**) Quantitative analysis of Ki67-positive cells in skin tissues. Data are presented as the mean ± SD (n = 3 independent skin samples from three different mice). * *p* < 0.05; *** *p* < 0.001 AGA compared to the Control group; P-MN compared to the B-MN group.

**Figure 8:**
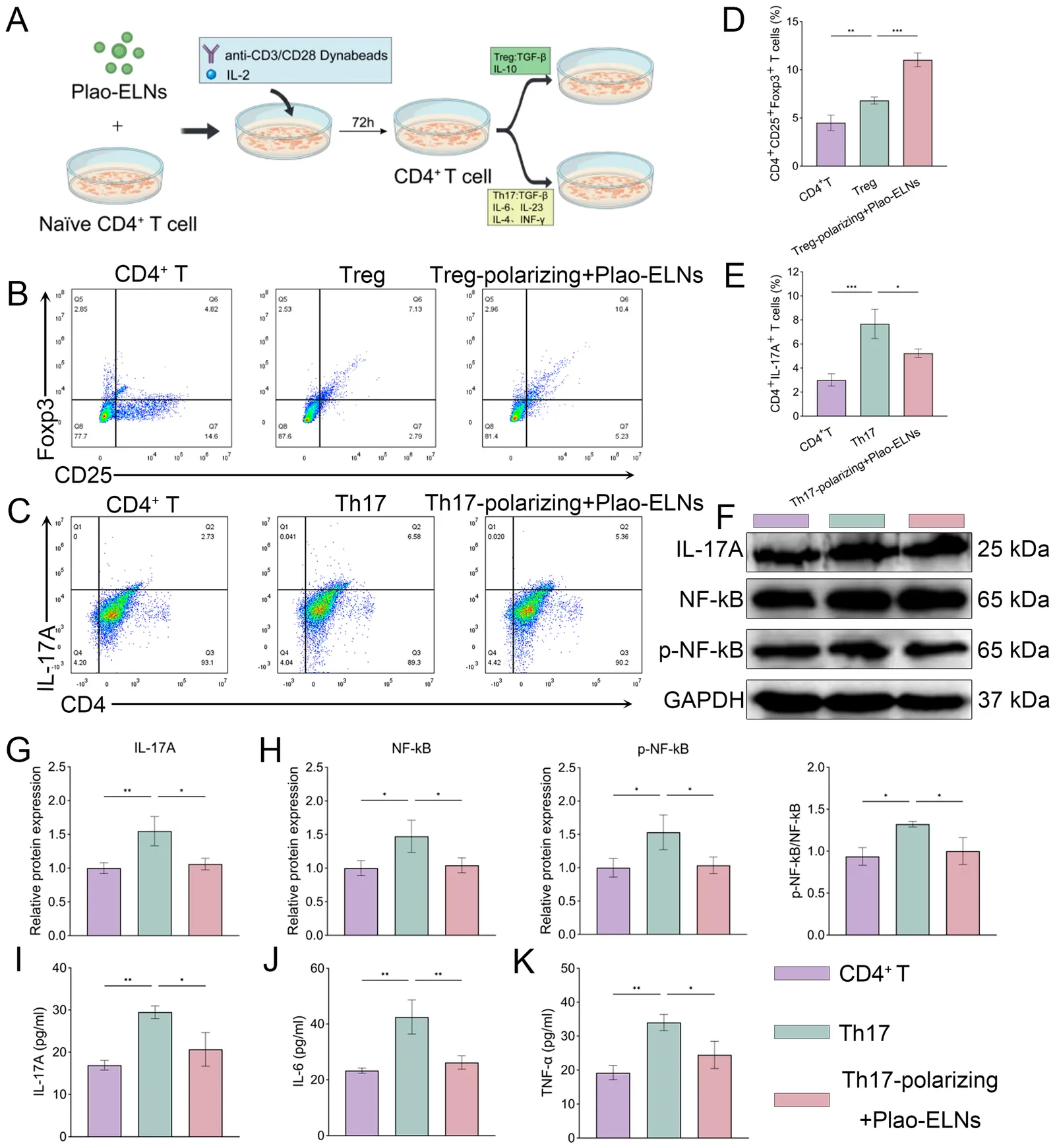
Plao-ELNs regulate CD4^+^ T cell differentiation and suppress the Treg/Th17 inflammatory axis in vitro. (**A**) Schematic illustration of CD4^+^ T cell differentiation into Treg and Th17 subsets. (**B**–**E**) Flow cytometric analysis and quantification of Treg and Th17 cell proportions under different treatments. (**F**) Western blot analysis of IL-17A, NF-κB, and p-NF-κB expression in CD4^+^ T cells. (**G**,**H**) Quantitative analysis of IL-17A, NF-κB, and p-NF-κB protein levels. (**I**–**K**) ELISA analysis of IL-17A, IL-6, and TNF-α levels in culture supernatants. Data are presented as the mean ± SD (n = 3 independent experiments). * *p* < 0.05; ** *p* < 0.01; *** *p* < 0.001.

**Figure 9:**
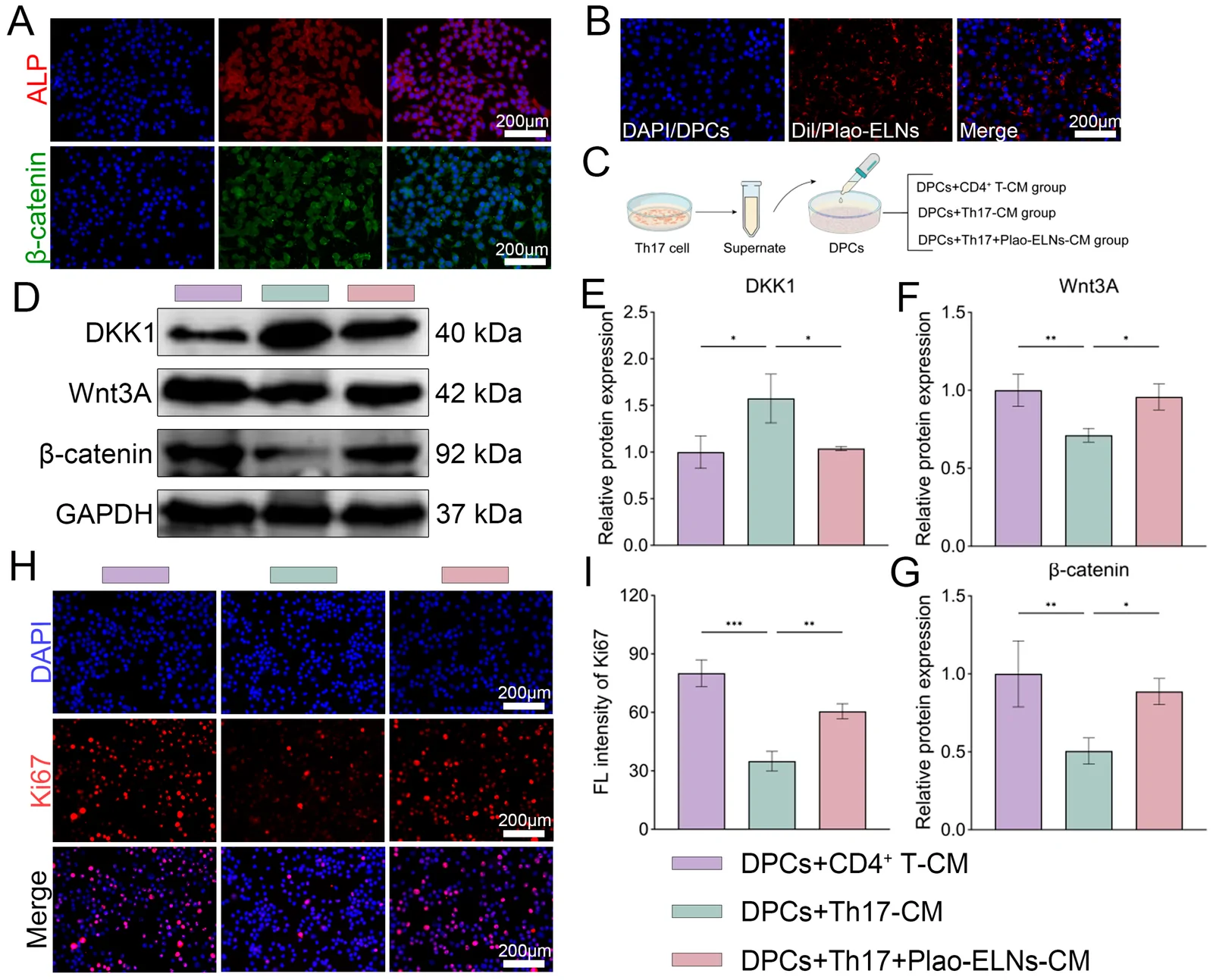
Plao-ELNs modulated the immune microenvironment to promote DPC proliferation via the DKK1/Wnt/β-catenin axis in vitro. (**A**) Identification of DPCs. (**B**) Uptake of Plao-ELNs by DPCs. (**C**) Schematic illustration of the conditioned medium (CM) experimental design: CM collected from Th17-polarized CD4^+^ T cells treated with or without Plao-ELNs was applied to DPCs to evaluate paracrine immune regulation. (**D**) Western blot analysis of DKK1, Wnt3A, and β-catenin expression in DPCs. (**E**–**G**) Quantitative analysis of DKK1, Wnt3A, and β-catenin protein levels. (**H**) Representative immunofluorescence images of Ki67 expression in DPCs. (**I**) Quantitative analysis of Ki67-positive DPCs. Data are presented as the mean ± SD (n = 3 independent experiments). * *p* < 0.05; ** *p* < 0.01; *** *p* < 0.001.

## Data Availability

All data supporting the findings of this study are available within the paper and its Supplementary Information. All data are available from the corresponding author on reasonable request.

## Supplementary Materials

The following supporting information can be downloaded at: https://www.mdpi.com/article/10.3390/biom16101505/s1, Figure S1: Low-magnification TEM fields of Plao-ELNs; Figure S2: The entire FTIR spectra of HA/MA/CP and HAMA/CP; Figure S3: TEM characterization of Plao-ELNs released from dissolved P-MNs; Figure S4: Histopathological Analysis of Major Organs; Table S1: Chemical characterization of Plao-ELNs using LC-MS/MS technology. Original Western blot images of Figure 6, Figure 7, Figure 8 and Figure 9.

## Abbreviations

The following abbreviations are used in this manuscript:

AGA	Androgenetic alopecia
Tregs	Regulatory T cells
PELNs	Plant-derived exosome-like nanoparticles
Plao-ELNs	Plao-derived exosome-like nanoparticles
MNs	Microneedles
NTA	Nanoparticle tracking analysis
LC-MS/MS	Liquid chromatography–tandem mass spectrometry
HAMA	Methacrylated Hyaluronic Acid
HA	Hyaluronic acid
MA	Methacrylic anhydride
CP	Cactus Polysaccharides
DPCs	Dermal papilla cells
P-MN	Plao-ELN-loaded Microneedles
PDMS	Polydimethylsiloxane
GO	Gene Ontology
KEGG	Kyoto Encyclopedia of Genes and Genomes
