# Supplementary material for: Platycladus orientalis Leaf-Derived Exosome-like Nanovesicles Delivered by Hydrogel Microneedles Restore Follicular Immune Homeostasis and Promote Hair Regeneration in Androgenetic Alopecia
Source: Biomolecules. 2026 Oct 15;16(10):1505. doi: 10.3390/biom16101505 (PMC13702671; doi:10.3390/biom16101505)
Supplement: Supplementary file 1 [file biomolecules-16-01505-s001.zip › Supplementary Figures.pdf]

# Supplementary Figures

## ***Platycladus orientalis leaf*-derived exosome-like nanovesicles delivered by hydrogel microneedles restore follicular immune homeostasis and promote hair regeneration in androgenetic alopecia**

**This file includes:**

Supplementary Text
Figure. S1 to S4
Table. S1

**Figure S1**

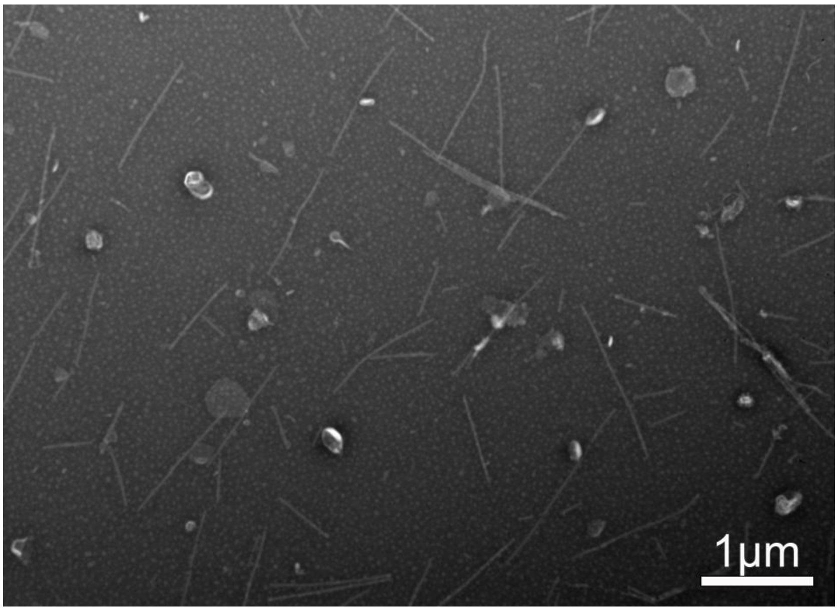


Figure S1. Low-magnification TEM fields of Plao-ELNs.

**Figure S2**

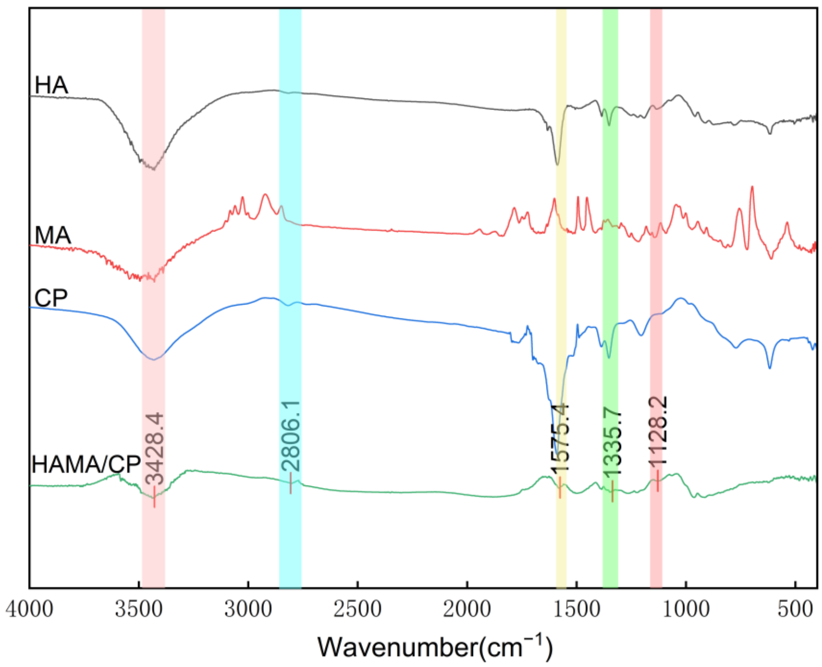


Figure S2. The entire FTIR spectra of HA/MA/CP and HAMA/CP

**Figure S3**

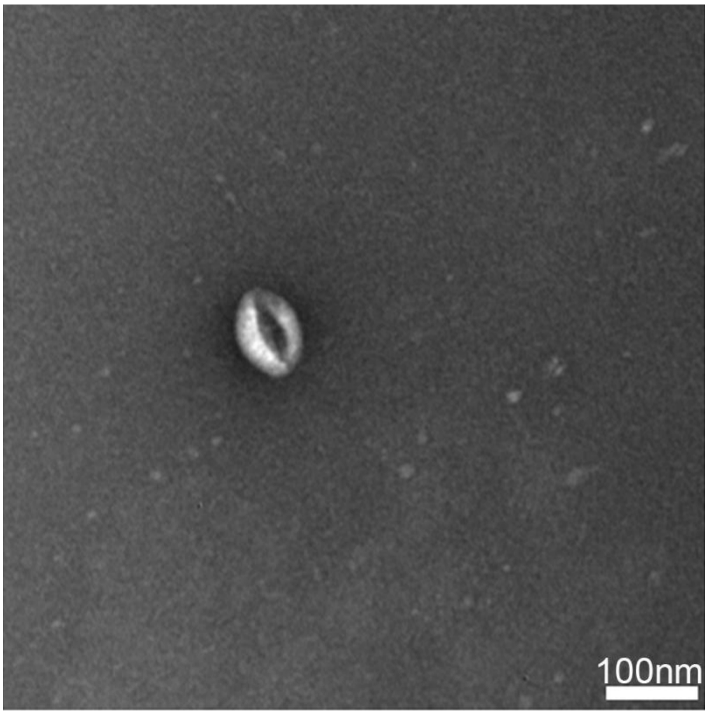


Figure S3. TEM characterization of Plao-ELNs released from dissolved P-MNs. Plao-ELNs were recovered from dissolved P-MN and examined by TEM. The released Plao-ELNs retained their characteristic vesicular morphology, with intact membrane structures and no obvious membrane disruption or extensive aggregation

**Figure S4**

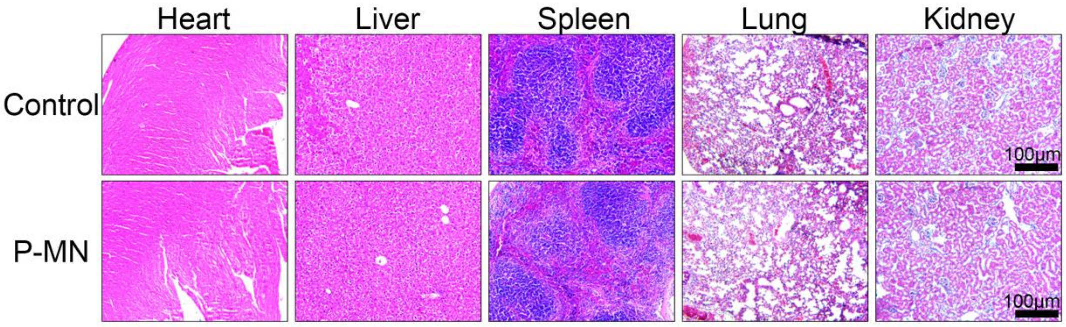


Figure S4. Histopathological analysis of major organs. Representative H&E-stained sections of the heart, liver, spleen, lungs, and kidneys from control mice and mice treated with P-MN. No significant tissue damage or pathological abnormalities were observed in any group.

**Table S1:**Chemical characterization of Plao-ELNs using LC-MS/MS technology

| NO. | Retention time (min) | Formula | Metabolites | PubChem | CAS | theoretical m/z | Observed m/z | Mass Error (ppm) | Area (Max.) |
|---|---|---|---|---|---|---|---|---|---|
| Positive model | | | | | | | | | |
| 1 | 18.657 | $C_8H_8O$ | Coumaran | 10329 | 496-16-2 | 103.05481 | 103.05439 | -4.075501 | 59425436.06 |
| 2 | 11.254 | $C_7H_8O_2$ | Orcinol | 10436 | 504-15-4 | 107.04973 | 107.04939 | -3.176094 | 4754757.59 |
| 3 | 0.817 | $C_5H_9NO_2$ | (R)-pyrrolidine-2-carboxylic acid | 8988 | 344-25-2 | 116.0706 | 116.0708 | 1.723089 | 730394470.7 |
| 4 | 1.163 | $C_5H_{11}NO_2$ | DL-Norvaline | | 760-78-1 | 118.08625 | 118.08642 | 1.439626 | 190332189.2 |
| 5 | 1.398 | $C_6H_5NO_2$ | Niacin | 938 | | 124.0393 | 124.03948 | 1.451153 | 14716193.97 |
| 6 | 0.849 | $C_7H_7NO_2$ | Trigonelline | 5570 | 535-83-1 | 138.05495 | 138.0548 | -1.086524 | 60433697.6 |
| 7 | 19.966 | $C_{11}H_{14}O_3$ | 3,4-Dimethoxycinnamyl alcohol | | 40918-90-9 | 177.09159 | 177.09084 | -4.235097 | 39984367.59 |
| 8 | 1.086 | $C_6H_{12}O_5$ | 1,5-Anhydrosorbitol | 64960 | | 187.05769 | 187.05773 | 0.213838 | 57216151.2 |
| 9 | 1.281 | $C_6H_{12}O_5$ | α-L-Rhamnose | 439710 | 6014-42-2 | 187.05769 | 187.05767 | -0.106919 | 35070219.76 |
| 10 | 0.99 | $C_6H_{12}O_6$ | Glucose | | 921-60-8 | 203.05261 | 203.05266 | 0.246242 | 223683573.5 |
| 11 | 6.705 | $C_9H_{18}N_2O_3$ | Alanyleucine | 96801 | 3303-34-2 | 203.13902 | 203.1391 | 0.393819 | 1500305.24 |
| 12 | 0.839 | $C_7H_{14}O_6$ | Methyl beta-D-Galactopyranoside | | 1824-94-8 | 217.06826 | 217.06792 | -1.566328 | 24029859137 |
| 13 | 15.598 | $C_{13}H_{20}O_3$ | Vomifoliol | 5280654 | 23526-45-6 | 225.14852 | 225.14884 | 1.421284 | 3524964.56 |
| 14 | 13.282 | $C_{13}H_{24}O_4$ | Megastigm-7-ene-3,5,6,9-tetraol | | 680617-50-9 | 227.16476 | 227.16417 | -2.597234 | 9327757.08 |
| 15 | 20.878 | $C_{15}H_{20}O_4$ | (±)-Abscisic acid | 5375199 | 14375-45-2 | 247.13346 | 247.133 | -1.861342 | 5219062.11 |
| 16 | 22.556 | $C_{15}H_{20}O_3$ | Rupestonic acid | 3081082 | 83161-56-2 | 266.17507 | 266.17271 | -8.866345 | 50220076.58 |
| 17 | 5.95 | $C_{10}H_{13}N_5O_5$ | Crotonoside | 65085 | 1818-71-9 | 284.09894 | 284.09913 | 0.668781 | 19521596.49 |
| 18 | 20.421 | $C_{15}H_{10}O_6$ | Scutellarein | 5281697 | 529-53-3 | 287.05501 | 287.05438 | -2.194701 | 544786787.6 |
| 19 | 28.529 | $C_{20}H_{30}O_3$ | Andrograpanin | | 82209-74-3 | 301.21679 | 301.21635 | -1.460742 | 9684688.79 |
| 20 | 10.715 | $C_{15}H_{16}O_8$ | Skimmin | | 93-39-0 | 325.09179 | 325.09213 | 1.045858 | 5158823.82 |
| 21 | 28.883 | $C_{20}H_{28}O_4$ | Marrubiin | 73401 | 465-92-9 | 333.20604 | 333.205 | -3.121192 | 130868082.7 |
| 22 | 13.841 | $C_{16}H_{18}O_8$ | 5-O-(E)-p-Coumaroylquinic acid | 6441280 | 5746-55-4 | 339.10744 | 339.10789 | 1.327013 | 33545887.02 |
| 23 | 12.973 | $C_{16}H_{22}O_7$ | Raspberry ketone glucoside | | 38963-94-9 | 349.12577 | 349.12589 | 0.343716 | 85732173.14 |
| 24 | 22.486 | $C_{20}H_{28}O_5$ | Ingenol | 3719 | 30220-46-3 | 371.18289 | 371.18308 | 0.511877 | 2032622.21 |
| 25 | 13.014 | $C_{16}H_{20}O_9$ | Cis-Ferulic acid 4-O-beta-D-glucopyranoside | | 94942-20-8 | 379.09995 | 379.10046 | 1.345292 | 169478371.3 |
| 26 | 20.898 | $C_{20}H_{26}O_6$ | Nodosin | 10248089 | 10391-09-0 | 385.16216 | 385.16233 | 0.441373 | 15713899.27 |

| | | | | | | | | | |
|---|---|---|---|---|---|---|---|---|---|
| 27 | 11.98 | C17H24O9 | Syringin | 5316860 | 118-34-3 | 390.17586 | 390.17618 | 0.820143 | 6189865.16 |
| 28 | 8.451 | C16H22O10 | Geniposidic acid | 443354 | 27741-01-1 | 397.11051 | 397.11123 | 1.813097 | 7839026.41 |
| 29 | 10.923 | C16H24O10 | Loganic acid | 89640 | 22255-40-9 | 399.12616 | 399.12629 | 0.325712 | 4389673.9 |
| 30 | 13.686 | C19H30O8 | Roseoside | | 54835-70-0 | 409.18329 | 409.18265 | -1.564091 | 1629481037 |
| 31 | 14.502 | C18H24O10 | Regaloside A | | 114420-66-5 | 423.12616 | 423.12651 | 0.827176 | 4684568.85 |
| 32 | 10.345 | C17H24O11 | Feretoside | 442433 | 27530-67-2 | 427.12108 | 427.12102 | -0.140475 | 55202914.68 |
| 33 | 11.151 | C17H24O11 | 10-Hydroxymajoroside | | 259753-12-3 | 427.12108 | 427.12263 | 3.628948 | 6660446.29 |
| 34 | 9.239 | C17H24O11 | Methyl deacetylasperulosidate | | 52613-28-2 | 427.12108 | 427.12241 | 3.113871 | 8319817.57 |
| 35 | 17.132 | C21H22O10 | Isohemiphloin | | 3682-2-8 | 457.11051 | 457.11152 | 2.209531 | 5160304.55 |
| 36 | 16.426 | C21H20O12 | 6-Hydroxyluteolin 7-glucoside | 185766 | 54300-65-1 | 465.10275 | 465.1038 | 2.257566 | 6778809.5 |
| 37 | 20.677 | C21H36O10 | A-D-Glucopyranoside | | 88700-35-0 | 471.22007 | 471.22041 | 0.721531 | 25803916.71 |
| 38 | 17.942 | C21H20O12 | Isoquercitrin | | 21637-25-2 | 487.08469 | 487.08521 | 1.067576 | 42257629.5 |
| 39 | 11.605 | C21H26O13 | Fabiatrin | | 18309-73-4 | 509.12656 | 509.12771 | 2.25877 | 2652765.15 |
| 40 | 0.844 | C18H32O16 | Raffinose | 439242 | 512-69-6 | 527.15825 | 527.15818 | -0.132787 | 94961714.37 |
| 41 | 20.255 | C26H32O11 | Matairesinoside | 486612 | 23202-85-9 | 543.18368 | 543.18382 | 0.25774 | 19400422.89 |
| 42 | 15.389 | C27H30O15 | Saponarin | 441381 | 20310-89-8 | 617.14769 | 617.14929 | 2.592572 | 4408495.94 |
| 43 | 16.426 | C26H28O16 | Quercetin 3-sambubioside | 5487635 | 83048-35-5 | 619.12695 | 619.12849 | 2.487374 | 11977559.15 |
| Negative model | | | | | | | | | |
| 1 | 7.274 | C6H6O2 | Catechol | 289 | 120-80-9 | 109.0295 | 109.0285 | -9.17183 | 8553426.88 |
| 2 | 0.871 | C4H6O5 | D-(+)-Malic acid | 92824 | 636-61-3 | 115.00313 | 115.00275 | -3.304258 | 108838832.7 |
| 3 | 6.822 | C7H6O4 | Protocatechuic acid | 72 | 99-50-3 | 153.01933 | 153.01864 | -4.509234 | 21283058.07 |
| 4 | 15.373 | C9H6O3 | Umbelliferone | 5281426 | 93-35-6 | 161.02442 | 161.02372 | -4.347167 | 11578090.85 |
| 5 | 14.761 | C9H8O3 | p-Coumaric acid | 637542 | 7400-08-0 | 163.04007 | 163.03926 | -4.968104 | 251803913.9 |
| 6 | 11.766 | C8H8O4 | 2,6-Dimethoxy-1,4-benzoquinone | 68262 | 530-55-2 | 167.03498 | 167.03427 | -4.250607 | 7744910.86 |
| 7 | 1.115 | C7H10O5 | Shikimic acid | 8742 | | 173.04555 | 173.04497 | -3.351719 | 225593418.9 |
| 8 | 21.215 | C8H8O2 | 4-Toluic acid | 7470 | 99-94-5 | 181.05063 | 181.05008 | -3.037824 | 27738249.42 |
| 9 | 29.156 | C12H24O3 | 3-Hydroxydodecanoic acid | 5312805 | 45162-48-9 | 215.16527 | 215.16501 | -1.208373 | 2675601.17 |
| 10 | 3.162 | C12H16O7 | Arbutin | 440936 | 497-76-7 | 271.08233 | 271.08241 | 0.295113 | 2569849.17 |
| 11 | 9.572 | C13H18O7 | Orcinol glucoside | | 21082-33-7 | 285.09798 | 285.09848 | 1.753783 | 2439851.52 |
| 12 | 21.194 | C16H12O7 | Nepetin | 5317284 | 520-11-6 | 315.05103 | 315.05135 | 1.015708 | 2411182.37 |
| 13 | 8.8 | C14H18O9 | Vanillic acid 4-beta-D-glucopyranoside | 14132337 | 32142-31-7 | 329.08781 | 329.08805 | 0.729289 | 23694472.19 |
| 14 | 6.25 | C14H18O9 | Phaseoloidin | | 118555-82-1 | 329.08781 | 329.08818 | 1.12432 | 12446270.5 |

| | | | | | | | | | |
|---|---|---|---|---|---|---|---|---|---|
| 15 | 11.245 | C16H26O8 | Jasminoside B | | 214125-04-9 | 345.15549 | 345.15567 | 0.521504 | 3093664.44 |
| 16 | 10.031 | C15H18O8 | 4-O-beta-Glucopyranosyl-cis-coumaric acid | 10604651 | 117405-48-8 | 371.09837 | 371.09873 | 0.970093 | 37797930.94 |
| 17 | 0.827 | C12H22O11 | Turanose | 5460935 | 547-25-1 | 377.08561 | 377.0857 | 0.238673 | 130352167.9 |
| 18 | 22.141 | C20H30O5 | 17-Hydroxyisolathyrol | | 93551-00-9 | 385.17873 | 385.1764 | -6.04914 | 11347231.44 |
| 19 | 14.179 | C17H24O10 | Verbenalin | 73467 | 548-37-8 | 387.12967 | 387.12963 | -0.103325 | 17160549.6 |
| 20 | 20.07 | C20H18O10 | Kaempferol 3-arabinofuranoside | 5748554 | 5041-67-8 | 417.08272 | 417.0838 | 2.589414 | 12763888.52 |
| 21 | 18.469 | C21H20O10 | Apigenin 5-O-glucoside | 14730806 | 28757-27-9 | 431.09837 | 431.09834 | -0.06959 | 6722847.52 |
| 22 | 19.841 | C21H20O10 | Apigenin 7-glucoside | | 578-74-5 | 431.09837 | 431.0986 | 0.533521 | 41884232.58 |
| 23 | 18.519 | C20H18O11 | Guaijaverin | 5481224 | 22255-13-6 | 433.07764 | 433.07857 | 2.147421 | 21013100.17 |
| 24 | 20.259 | C21H24O10 | Phlorizin | | 60-81-1 | 435.12967 | 435.13013 | 1.057156 | 15870722.25 |
| 25 | 18.101 | C21H20O11 | 2-(3,4-dihydroxyphenyl)-7-(beta-D-glucopyranosyloxy)-8-hydroxy-4H-1-benzopyran-4-one | | 925701-05-9 | 447.09329 | 447.09355 | 0.581534 | 5519341.55 |
| 26 | 19.596 | C21H20O11 | Quercitrin | 5280459 | 522-12-3 | 447.09329 | 447.09406 | 1.722236 | 7272372213 |
| 27 | 16.11 | C21H22O12 | Taxifolin 3'-O-glucoside | | 31106-05-5 | 465.10385 | 465.10449 | 1.376037 | 5309327.95 |
| 28 | 17.696 | C21H30O9 | β-D-Glucopyranosyl abscisate | 46173811 | 21414-42-6 | 471.18718 | 471.18802 | 1.782731 | 17973251.8 |
| 29 | 18.91 | C26H36O11 | Secoisolariciresinol monoglucoside | | 63320-67-2 | 523.21849 | 523.21923 | 1.414323 | 174197458 |
| 30 | 17.911 | C26H32O12 | Nortracheloside | | 33464-78-7 | 535.1821 | 535.18316 | 1.980634 | 12445068.8 |
| 31 | 1.276 | C18H32O16 | 1-Kestose | 440080 | 470-69-9 | 539.13844 | 539.13905 | 1.131435 | 19150138.42 |
| 32 | 17.932 | C26H28O15 | Leucoside | 44566720 | 27661-51-4 | 579.13554 | 579.1366 | 1.830314 | 33231606.18 |
| 33 | 19.582 | C28H36O13 | Episyringaresinol 4'-O-beta-D-glncopyranoside | | 137038-13-2 | 625.21379 | 625.2133 | -0.783732 | 3659841.7 |
| 34 | 11.328 | C16H20O9 | Trans-ferulic acid-4-beta-glucoside | | 537-98-4 | 711.21419 | 711.21568 | 2.095009 | 3739825.37 |
| 35 | 15.35 | C32H42O16 | Pinoresinol Diglucoside | 174003 | 63902-38-5 | 727.24548 | 727.24631 | 1.141293 | 6134411.02 |
