# Supplementary material for: Platycladus orientalis Leaf-Derived Exosome-like Nanovesicles Delivered by Hydrogel Microneedles Restore Follicular Immune Homeostasis and Promote Hair Regeneration in Androgenetic Alopecia
Source: Biomolecules. 2026 Oct 15;16(10):1505. doi: 10.3390/biom16101505 (PMC13702671; doi:10.3390/biom16101505)
Supplement: Supplementary file 1 [file biomolecules-16-01505-s001.zip › original Western blot images.pdf]

Figure 6

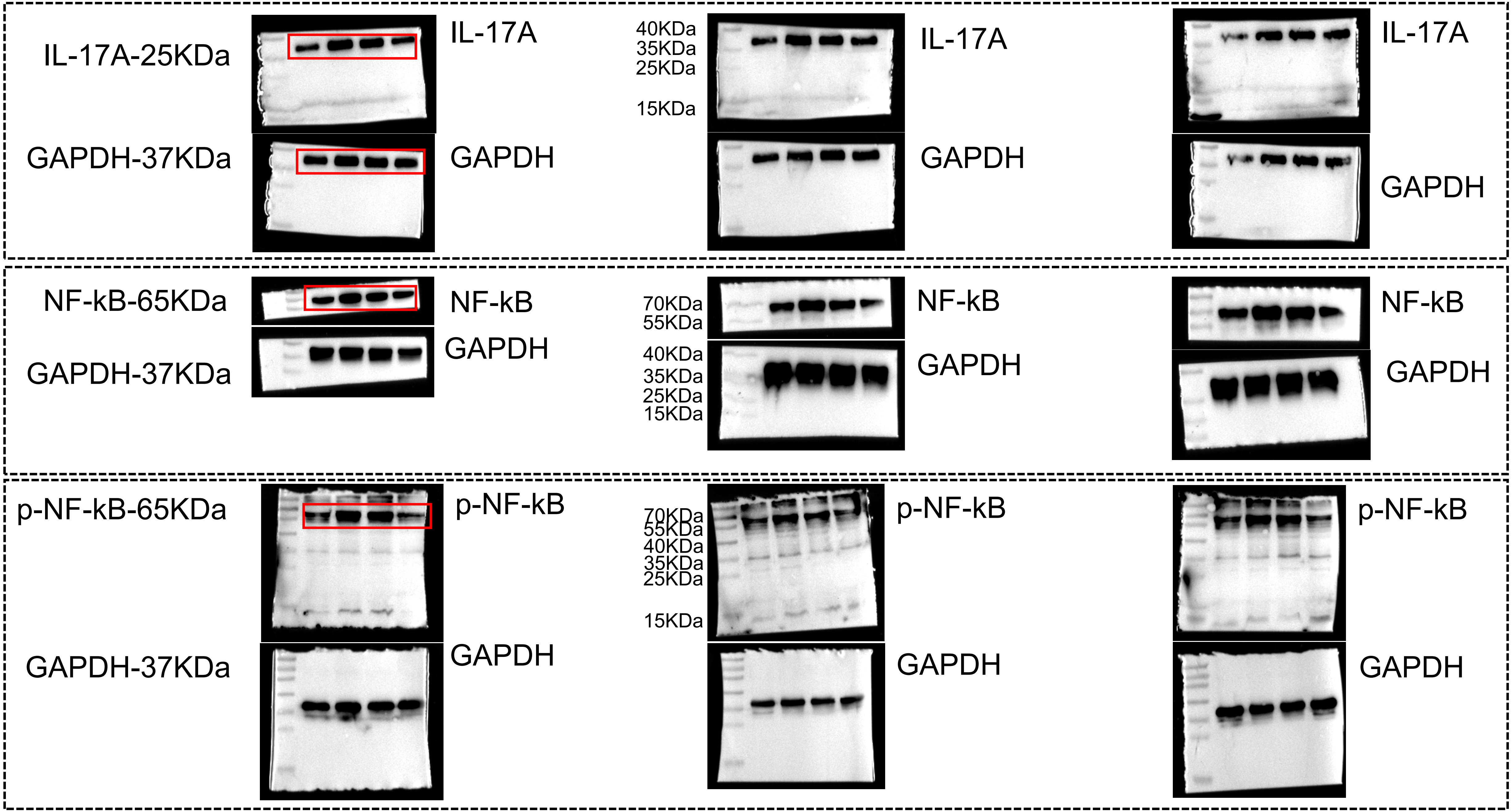
IL-17A-25KDa
IL-17A
GAPDH-37KDa
GAPDH
40KDa
35KDa
25KDa
15KDa
IL-17A
GAPDH
IL-17A
GAPDH
NF-kB-65KDa
NF-kB
GAPDH-37KDa
GAPDH
70KDa
55KDa
NF-kB
40KDa
35KDa
25KDa
15KDa
GAPDH
NF-kB
GAPDH
p-NF-kB-65KDa
p-NF-kB
GAPDH-37KDa
GAPDH
70KDa
55KDa
40KDa
35KDa
25KDa
15KDa
p-NF-kB
GAPDH
p-NF-kB
GAPDH

Figure 7

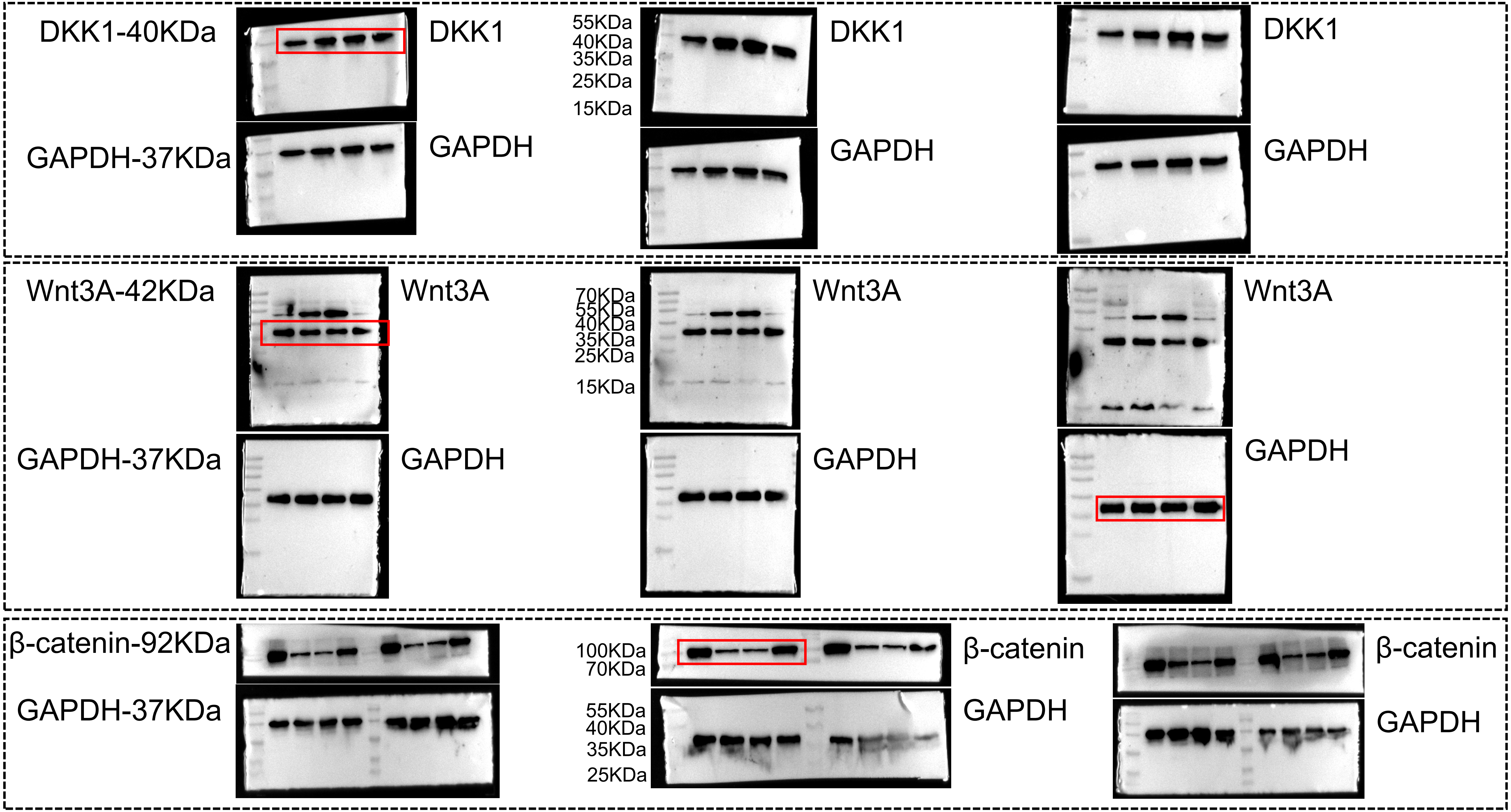
DKK1-40KDa
DKK1
GAPDH-37KDa
GAPDH
55KDa
40KDa
35KDa
25KDa
15KDa
DKK1
GAPDH
DKK1
GAPDH
Wnt3A-42KDa
Wnt3A
GAPDH-37KDa
GAPDH
70KDa
55KDa
40KDa
35KDa
25KDa
15KDa
Wnt3A
GAPDH
Wnt3A
GAPDH
β-catenin-92KDa
GAPDH-37KDa
100KDa
70KDa
55KDa
40KDa
35KDa
25KDa
β-catenin
GAPDH
β-catenin
GAPDH

Figure 8

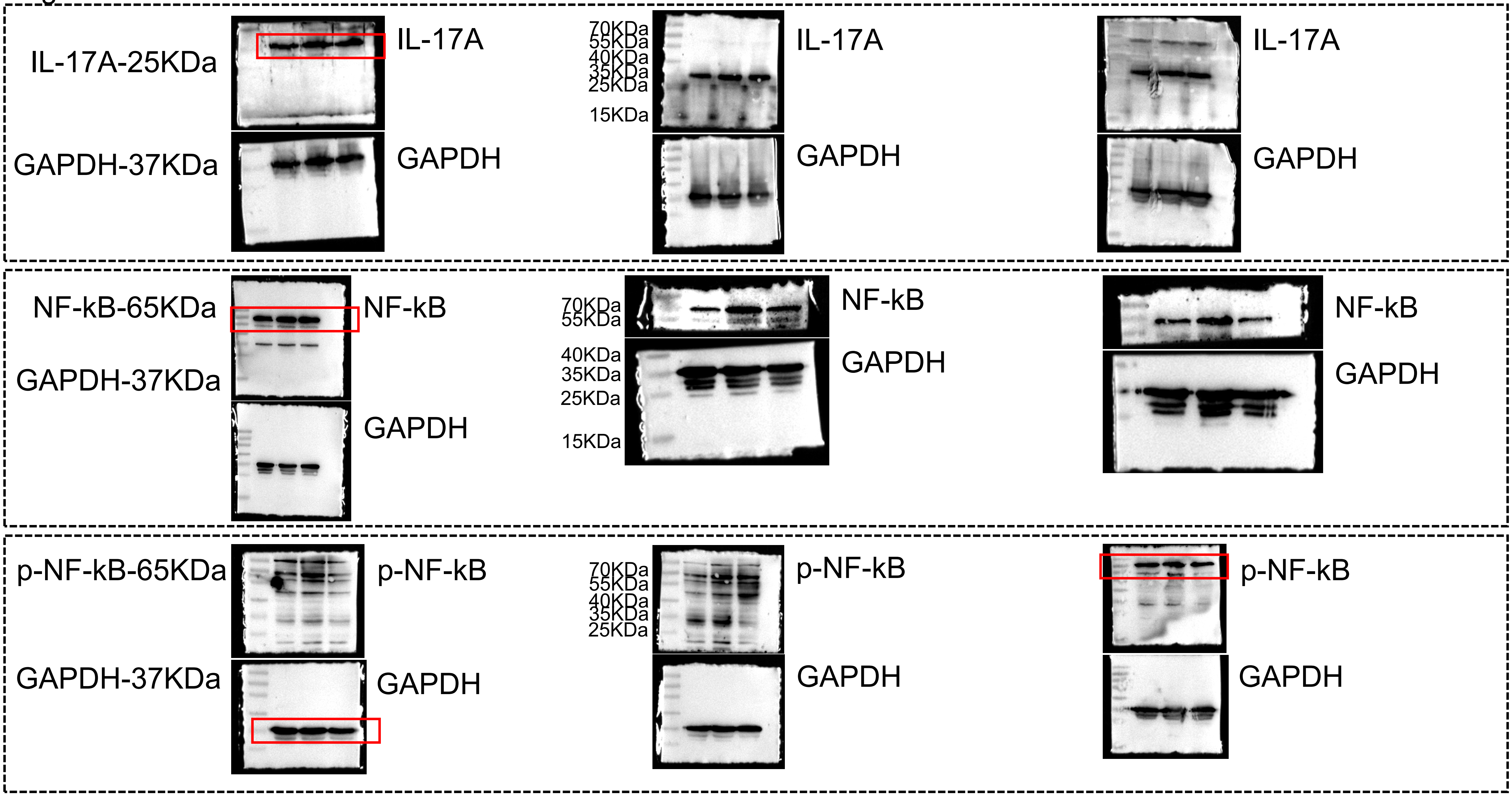

IL-17A-25KDa
IL-17A
GAPDH-37KDa
GAPDH
70KDa
55KDa
40KDa
35KDa
25KDa
15KDa
IL-17A
GAPDH
IL-17A
GAPDH
NF-kB-65KDa
NF-kB
GAPDH-37KDa
GAPDH
70KDa
55KDa
NF-kB
40KDa
35KDa
25KDa
15KDa
GAPDH
NF-kB
GAPDH
p-NF-kB-65KDa
p-NF-kB
GAPDH-37KDa
GAPDH
70KDa
55KDa
40KDa
35KDa
25KDa
p-NF-kB
GAPDH
p-NF-kB
GAPDH

Figure 9

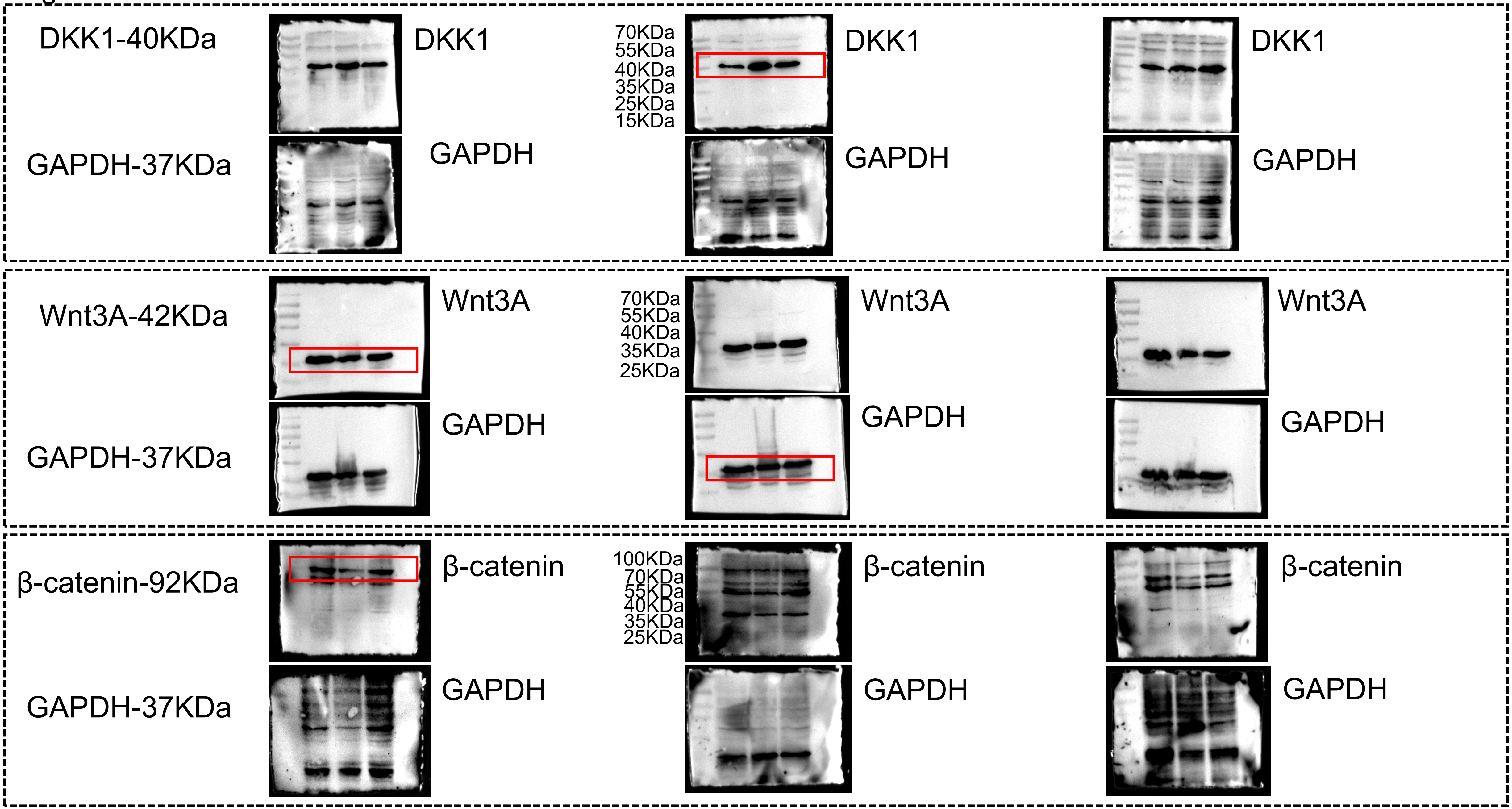
DKK1-40KDa
DKK1
70KDa
55KDa
40KDa
35KDa
25KDa
15KDa
DKK1
DKK1
GAPDH-37KDa
GAPDH
GAPDH
GAPDH
Wnt3A-42KDa
Wnt3A
70KDa
55KDa
40KDa
35KDa
25KDa
Wnt3A
Wnt3A
GAPDH-37KDa
GAPDH
GAPDH
GAPDH
β-catenin-92KDa
β-catenin
100KDa
70KDa
55KDa
40KDa
35KDa
25KDa
β-catenin
β-catenin
GAPDH-37KDa
GAPDH
GAPDH
GAPDH
